# Lactoferrin thermal stabilization and iron(II) fortification through ternary complex fabrication with succinylated sodium caseinate

**DOI:** 10.1016/j.fochx.2024.101498

**Published:** 2024-05-23

**Authors:** Yunan Huang, Tiantian Lin, Younas Dadmohammadi, Yanhong He, Waritsara Khongkomolsakul, Claire Elizabeth Noack, Alireza Abbaspourrad

**Affiliations:** Department of Food Science, College of Agriculture and Life Sciences, Cornell University, Ithaca, NY, USA

**Keywords:** Lactoferrin, Sodium caseinate, Succinylation, Electrostatic complexation, Iron(II) delivery, Thermal stability

## Abstract

A thermally stable co-delivery system for lactoferrin (LF) and iron(II) was developed to address iron deficiency anemia. Complexes were formed between LF, succinylated sodium caseinate (S.NaCas) and FeSO_4_ with high yield (∼85%). LF-S.NaCas-Fe complexes achieved loading capacities for iron(II) between 2.5 and 12 mg g^–1^and LF loading capacities between 250 and 690 mg g^−1^, depending upon initial Fe^2+^ concentrations and LF ratios. The LF-S.NaCas complex mixtures appeared as smooth cubic particles in SEM, and gradually aggregated to amorphous particles as th iron(II) concentration increased due to iron-facilitated cross-linking. The complexation significantly improved LF thermal stability and addressed the poor solubility of iron(II) under neutral pH. After thermal treatment (95 °C, 5 min), the rehydrated complexes retained 68%–90% LF, with <10% iron(II) release. Circular dichroism spectra showed the secondary structure of the complexed LF was well retained during thermal treatment. This thermally stable system showed great potential in LF thermal protection and iron(II) fortification.

## Highlights

• Lactoferrin and succinylated sodium caseinate form the hetero-protein complexes.
• The complexes are formed through electrostatic interaction at pH 4.5 and 5.0.
• Iron(II) works as a cross-linker in the complex formation and gets encapsulated.
• Iron(II) changes the complex micro-structure and morphology through cross-linking.
• Complexation improves lactoferrin thermal stability and Fe^2+^ solubility at pH 7.0.

## 1 Introduction

Iron is an essential mineral for the human body, and inadequate iron intake leads to iron deficiency anemia (IDA), causing fatigue, weakness, and impaired cognitive function (Kumar, Arnipalli, Mehta, Carrau, & Ziouzenkova, 2022; Means, 2020). IDA plagues ∼25% of the global populations, particularly in infants, young children, pregnant women, and females with heavy menses (Shubham et al., 2020). To combat IDA, many iron-fortified foods have been developed to improve iron uptake, the most common are iron-fortified cereals and flour (Cardoso, Fernandes, Gonzaléz-Paramás, Barros, & Ferreira, 2019; Shubham et al., 2020). However, there are challenges associated with iron fortification that need to be addressed. First, in neutral pH iron(II) oxidizes and precipitates, triggering undesirable changes in color and flavor, and compromising its bioavailability. Meanwhile, free iron(II) causes diarrhea and other gastrointestinal (GI) side effects. Therefore, it is necessary to construct delivery systems for the stable encapsulation and delivery of iron(II).

Bovine lactoferrin (LF) is an iron-binding glycoprotein with two specific iron-binding sites in each molecule, located in its C- and N-lobes (Mikulic et al., 2020). LF possesses several beneficial health attributes including antioxidant, anti-bacterial and anti-inflammatory properties. LF also facilitates iron(II) absorption (Zhao et al., 2022) and improves gut health (Jenssen & Hancock, 2009). With a high affinity to iron(III) (*K*_d_ ∼ 10^−20^ M) (Rosa, Cutone, Lepanto, Paesano, & Valenti, 2017), LF can bind to unabsorbed luminal iron in the distal gut (pH 6.5–7.5), reducing the pro-oxidant effect of iron(II) on the mucosa (Bielecka, Cichosz, & Czeczot, 2022). Thus, iron(II) delivery with LF is a favorable approach to increasing dietary iron intake with reduced undesired flavors and GI irritation. However, one factor that limits the application of LF is its thermal instability, especially in neutral pH (Bokkhim, Bansal, GrØndahl, & Bhandari, 2013). Thermal denaturation of LF begins at 61 °C at pH 7.0 in the aqueous solution (Bengoechea, Peinado, & McClements, 2011). Heat treatment is often used in food processing to ensure food safety, flavor maintenance and shelf-life extension. Therefore, improving the thermal stability of LF is crucial for its use within the food industry.

Electrostatic complexation, or coacervation is a phase separation phenomenon between two or more oppositely charged macromolecules under specific conditions, such as temperature, pH, ionic strength, and mixing ratio. When the net charges of the biopolymers achieve electrostatic neutralization, the complex is formed and the biopolymer complex precipitates from solution (Zheng et al., 2020). These complex systems have shown good biocompatibility, are considered clean label and are made via a simple preparation process, thus these electrostatic complexation systems are being used in the delivery of biological macromolecules such as proteins and polysaccharides, and as delivery vehicles for other small molecules (Chapeau et al., 2016). Moreover, electrostatic complexation has been proven to improve the thermal stability of LF (Lin et al., 2022, Lin et al., 2023). Different from most proteins, LF possesses a rather high isoelectric point (pI,∼8.0), so is positively charged in a wide pH range, which makes it possible to form complexes with other negatively charged proteins or polysaccharides (Lin et al., 2023; F. Liu, Zhang, Li, McClements, & Liu, 2018).

Sodium caseinate (NaCas) is the sodium salt of casein, which is the main protein (80 *w*/w%) in bovine milk (Thomar & Nicolai, 2015). Its low pI (4.0–4.5) makes it a suitable candidate to form hetero-protein complexes with LF. NaCas also has considerable thermal stability (Wu, Liu, Liu, & Wang, 2017) and could potentially protect LF from thermal denaturation. NaCas also shows a mineral-binding property and can chelate and stabilize iron ions with specific binding sites, including phosphate and carboxylic groups (Shilpashree, Arora, Kapila, & Sharma, 2018; Sugiarto, Ye, & Singh, 2009). However, from preliminary screening, the complexes between LF and NaCas had low turbidity and poor yields at different pH levels (**Fig. S1A**), due to the low negative charges (zeta potential > −5 mV) of NaCas in aqueous solution. Therefore, protein modification was proposed to introduce additional negative charges into NaCas and enhance the electrostatic interaction strength with LF.

Protein succinylation is a chemical modification procedure wherein succinic acid moieties are covalently attached to the lysine in protein molecule (Agarwal et al., 2020; Shilpashree, Arora, Chawla, Vakkalagadda, & Sharma, 2015), resulting in the replacement of positively charged -NH_2_ group with the negatively charged succinyl group (-CH_2_-COO^−^), thereby enhancing the negative charges on the protein surface. Except for charge modification, succinylation also shows improvement in the iron binding capacity of NaCas (Agarwal et al., 2020; Shilpashree, Arora, Sharma, & Singh, 2015). It is reported that the maximum iron(II) solubility in 1 *w*/*v*% NaCas was 5.4 mM, and with more iron(II) added, NaCas would precipitate from solution. However, after succinylation, the saturated iron(II) binding concentration in 1 w/v% succinylated NaCas (S.NaCas) was increased to 7.4 mM, and no protein precipitate after reaching saturation (Shilpashree, Arora, Sharma, & Singh, 2015).

We hypothesized that S.NaCas would have sufficient negative charges to form hetero-protein complexes with LF through electrostatic interactions and that due to the enhanced chelation between iron and S.NaCas, the ternary LF-S.NaCas-Fe complex would form and achieve efficient and stable co-delivery of LF and Fe.

Herein, we carried on the succinylation of NaCas and examined the optimal complex formation of LF and S.NaCas to achieve a high yield (> 50%). The effect of iron(II) concentration on complex formation was investigated to explore the interactions between iron and protein components, and LF and Fe^2+^ loading was optimized simultaneously. The microstructure of the complexes was investigated, and the thermal stability of encapsulated LF was studied during thermal treatment through turbidity, particle size, and protein secondary structure change, as well as the LF retention ratio. The successful realization of this work would present a promising Fe^2+^ delivery system, enrich our understanding of LF thermal stability, and provide a potential co-delivery system in iron-fortified foods and protein-functional foods.

## 2 Materials and methods

### 2.1 Materials

Bovine LF (Bioferrin 2000; Iron >15 mg/100 g) was purchased from Glanbia Nationals, Inc. (Fitchburg, WI, USA). Casein sodium salt from bovine milk (NaCas), succinic anhydride, ferrous sulfate heptahydrate, and l-ascorbic acid were purchased from Sigma-Aldrich (St. Louis, MO, USA). Sodium hydroxide, hydrochloric acid, trifluoroacetic acid (HPLC grade), and acetonitrile (HPLC grade) were purchased from Fisher Scientific (Hampton, NH, USA). Ammonium acetate was purchased from Research Products International (Mt Prospect, IL, USA). Ferrozine was purchased from Cayman Chemical Company (Ann Arbor, MI, USA). Milli-Q water (18.2 MΩ/cm) from the Millipore purification system (Sigma, MA, U.S.A.) was used to prepare all aqueous solutions.

### 2.2 Methods

#### 2.2.1 Zeta-potential and particle size

Particle size (*Z*-average), particle size distribution (PSD) based on intensity, polydispersity index (PDI), and zeta-potential of samples were measured using a Zetasizer (Malvern Nano-ZS90, U.K). Particle size measurements were conducted using dynamic light scattering (DLS), using disposable 1 cm path square cuvettes with backscattering angle 173° and refractive index 1.33 for carrying material (water) and 1.45 for measuring material (protein). The zeta-potential was measured with disposable folded capillary cells under Smouluchwski mode at 150 V. All measurements were performed at 25 °C in triplicates with at least 11 runs for each measurement.

#### 2.2.2 Turbidity

The turbidity of sample mixtures was evaluated by transmittance by UV–Vis light spectrophotometer (UV-2600, SHIMADZU Co., Japan) at 600 nm. Milli-Q water was used as the blank (100% transmittance). The turbidity (T) was then calculated by the following equation (Zheng et al., 2020):

(1) $$\mathrm{Turbidity}\;\left(T\right)=-\ln\frac{I}{I_{0}}\;$$

where I_0_ and I are the transmittance of blank (Milli-Q water) and the sample, respectively. All samples were measured at room temperature.

#### 2.2.3 Fourier transform infrared (FT-IR) spectroscopy

The FT-IR spectra of different sample powers were collected on a Shimadzu IRAffinity-1S (Shimadzu Corp., Japan). The spectra were scanned 400 to 4000 cm^−1^ with a resolution of 4 cm^−1^ and 64 scans for each measurement. A background spectrum was collected first before each sample.

#### 2.2.4 Circular dichroism (CD) spectroscopy

The CD spectra of protein and complex solutions were collected by JASCO-1500 CD Spectrometer (JASCO, Japan) to analyze the protein secondary structure. All samples were diluted to 0.02 mg mL^−1^ before measurement to avoid signal gain. Scan range was 190–260 nm and 10 mm path length quartz cells were used for the measurements. The secondary structure information of the spectra was analyzed on DichroWeb (Miles, Ramalli, & Wallace, 2022).

#### 2.2.5 Scanning electron microscope (SEM)

The microstructures and morphologies of proteins and complexes were analyzed by emission scanning electron microscope (SEM, Zeiss Gemini 500, Jena, Germany). The element composition was further analyzed by the energy-dispersive X-ray (EDX) technology.

For powder samples, small amount of fine powder was splashed on a pin stab with carbon tape and coated with Au/Pt in the sputter coater (Denton Desk V, NJ, USA); for solution samples, around 10 μL solution was evenly applied on the pin stab with carbon tape and vacuum dried overnight. Carbon coating was used for solution samples and for further EDX analysis. All samples were scanned and photographed with the high efficiency secondary electron detector with a 20.0 μm aperture with the HE-SE2 signal (Lin et al., 2023). The accelerating voltage was 1–3 kV for imaging and 20 kV for EDX analysis. EDX analysis was conducted with the AZtec software (Oxford instrument, UK).

### 2.3 Succinylation of sodium caseinate

#### 2.3.1 NaCas succinylation

NaCas succinylation was carried out as described (Shilpashree, Arora, Chawla, et al., 2015) with slight modifications. To be specific, 10 g NaCas was dissolved in 100 mL milli-Q water with constant stirring at 37 °C. The pH was adjusted to 8.0 using 2 M NaOH. Subsequently, a specific amount dependent on experiment (0.5–2 g) of dry succinic anhydride (SA) was added into the NaCas solution and the pH was readjusted to 8.0. Then the mixture was stirred for 1 h at 37 °C, during which the pH was monitored and kept at 8.0. Protein recovery was then conducted by precipitation at pH 3.5–4.0 using 2 M HCl. The precipitate was collected by centrifuging at 5000*g* for 20 min at 25 °C and re-suspended in 100 mL of milli-Q water. The precipitate was washed for 1 h with constant stirring at room temperature and centrifuged again. This process was repeated 4 times to remove extra SA and salts. The final pellet was redispersed in milli-Q water and the pH adjusted to 7.0 using 2 M NaOH. Then the solution was frozen overnight under −80 °C and freeze-dried (Labcono, Kansas, MO, USA), and the dry product was then ground by mortar and pestle to a fine powder and stored at −18 °C.

#### 2.3.2 Degree of succinylation (DS)

The DS of S.NaCas was analyzed using the ninhydrin method (Shilpashree, Arora, Chawla, et al., 2015). The absorbance of samples was measured by UV–Vis spectrophotometer (UV-2600, SHIMADZU Co., Japan) at 570 nm using 1 cm path-length quartz cuvettes and the DS of S.NaCas was calculated with the following equation:

(2) $$\mathrm{Degree of succinylation}\;\left(\mathrm{DS},\%\right)=\frac{A_{0}-A_{1}}{A_{0}}\times 100\;$$

where A_0_ and A_1_ are the absorbance of native NaCas and S.NaCas, respectively (Pan et al., 2019).

### 2.4 Complex formation and characterization

#### 2.4.1 Strength of electrostatic interaction (SEI) between LF and S.NaCas

SEI between two oppositely charged biopolymers was expressed by the product of zeta-potential absolute values of the two polymers as a function of pH (Weinbreck, Tromp, & de Kruif, 2004). SEI provides a good basis for choosing the optimal pH for electrostatic complexation. To be specific, LF and S.NaCas were dissolved at 2 mg mL^−1^ in Milli-Q water separately, and the pH of the solutions was adjusted from 2.0 to 10.0 with 0.5 pH unit intervals using 1 M HCl or NaOH and their zeta-potential was measured as described in Section 2.2.1. Then the SEI value under each pH was calculated by the following equation:

(3) $${\mathrm{SEI}}_{\mathrm{pH}}=|\mathrm{ZP}\left(\mathrm{LF}\right)\times \mathrm{ZP}\left(S.\mathrm{NaCas}\right)|\;$$

where ZP(LF) and ZP(S.NaCas) were the zeta-potential of LF and S.NaCas at each pH.

#### 2.4.2 Preparation of LF-S.NaCas/-Fe complex

LF and S.NaCas stock solutions (10 mg mL^−1^) were prepared in Milli-Q water with magnetic stirring for 30 min at room temperature and stored overnight at 4 °C for full hydration. FeSO_4_ stock solution (1 M) was freshly prepared before use to avoid iron oxidation. All stock solutions were allowed to return to room temperature (25 °C) before use. The LF-S.NaCas complex was prepared with pH-first method, that is, the pH of LF and S.NaCas solutions were adjusted to the target pH separately with 1 M NaOH or HCl first, and then the two solutions were mixed at different LF to S.NaCas volume ratios from 4:1 to 1:4. For LF-S.NaCas-Fe complexes, FeSO_4_ stock solution was then added into the mixture in varying amounts. The pH of the mixtures was confirmed within the range of the target pH ± 0.2 after mixing, then the mixtures were shaken at 200 rpm for 30 min. All samples were prepared at room temperature.

The complex mixtures were then centrifuged at 15,000*g* for 15 min at 4 °C. The pellets were collected and frozen overnight at −18 °C, and then freeze-dried. The freeze-dried products were then ground to a fine powder with a mortar and pestle and stored at −18 °C for further characterization.

#### 2.4.3 Complex yield, LF encapsulation efficiency (EE) and loading capacity (LC)

The complex yield was calculated based on the freeze-dried complex mass by the following equation:

(4) $$\mathrm{Yield}\;\left(\%\right)=\frac{\mathrm{weight of freeze}-\mathrm{dried complex}}{\mathrm{weight of tota}l\;\mathrm{mass added in complex mixture}}\times 100\;$$

LF quantification was conducted using HPLC as described (Lin et al., 2023). All samples were filtered with 0.22 μm PVDF syringe filters before loading to remove any insoluble materials.

LF EE (%) was indirectly evaluated by measuring the free LF concentration by HPLC in the supernatant after centrifuging, while the LF LC (mg g^−1^) was analyzed by redispersing the 20 mg of the complex powder in 10 mL of Milli-Q water (pH was adjusted to 7.0 to fully dissolve complex), then measuring the LF concentration in the redispersed complex solution by HPLC. LF EE and LC were calculated by the following equations where [LF] is the concentration of lactoferrin:

(5) $$\mathrm{LF}\;\mathrm{EE}\;\left(\%\right)=\frac{\left[\mathrm{LF}\right]\;\mathrm{complex mixtu}re-\left[\mathrm{LF}\right]\;\mathrm{supernatant}}{\left[\mathrm{LF}\right]\;\mathrm{complex mixture}}\times 100\;$$

(6) $$\mathrm{LF}\;\mathrm{LC}\;\left(\mathrm{mg}\;g^{-1}\right)=\frac{\left[\mathrm{LF}\right]\;\mathrm{redispersed complex}\times \mathrm{volume of solution}}{\mathrm{weight of redispersed complex}}\;$$

#### 2.4.4 Fe EE and LC

The ferrozine method was used to quantify the iron content in the complexes (Nielsen, 2017). FeSO_4_ standard solutions (0–0.5 mM) were prepared, and standard curves were established. The absorbance of the standards and samples were measured as per ferrozine method and the Fe concentration in samples was calculated based on the standard curve.

Fe EE and LC were based upon the Fe concentration in supernatant and the redispersed complex solution with the following equations:

(7) $$\mathrm{Fe}\;\mathrm{EE}\;\left(\%\right)=\frac{\left[\mathrm{Fe}\right]\;\mathrm{complex mixture}-\left[\mathrm{Fe}\right]\;\mathrm{supernatant}}{\left[\mathrm{Fe}\right]\;\mathrm{complex mixture}}\times 100\;$$

(8) $$\mathrm{Fe}\;\mathrm{LC}\;\left(\mathrm{mg}\;g^{-1}\right)=\frac{\left[\mathrm{Fe}\right]\;\mathrm{redispersed complex}\times \mathrm{volume of solution}\times \mathrm{Mw}}{\mathrm{weight of redispersed complex}}\;$$

where [Fe] is iron concentration and Mw is the molecular weight of Fe (56 g mol^−1^).

### 2.5 Thermal study of LF-S.NaCas-Fe complex

For thermal treatment, complexes were redispersed 2 mg mL^−1^ in Milli-Q water and adjusted to target pH levels between 3.0 and 7.0. Complex suspensions (2 mL) were transferred to screw-capped test tubes and placed in a 95 °C water bath once temperature equilibrium was reached, they were allowed to sit for 5 min. The samples were then cooled in an ice bath immediately until the sample reached room temperature.

The turbidity, particle size, zeta-potential, CD spectra, LF retention, and Fe release were then analyzed for all samples. LF retention (%) was calculated as following equation:

(9) $$\mathrm{LF}\;\mathrm{retention}\;\left(\%\right)=\frac{\left[LF\right]\;\mathrm{after heating}}{\left[\mathrm{LF}\right]\;\mathrm{before heating}}\times 100\;$$

The released Fe was measured from the ultrafiltrate of heated samples with centrifugal filter units (3 K MWCO, 8000 *g*, 5 min) and Fe release (%) was calculated by the following equation:

(10) $$\mathrm{Fe}\;\mathrm{release}\;\left(\%\right)=\frac{\;\left[\mathrm{Fe}\right]\;\mathrm{ultrafiltrate}}{\left[\mathrm{Fe}\right]\;\mathrm{total}}\times 100\;$$

### 2.6 Statistical analysis

All measurements were performed in duplicates or triplicates and data were reported as the mean ± standard deviations. Significant difference between the values was analyzed by one way analysis of variance (ANOVA) and comparison between means was evaluated by Student's *t*-test (*p* < 0.05) using JMP pro 16 software.

## 3 Results and discussion

### 3.1 Succinylation of NaCas

Succinic anhydride (SA) reacts with the ε-amino group of lysine and replaces it with a negatively charged succinyl group which imparts an overall more negative charge to NaCas. Casein contains 8.5 g of lysine for every 100 g of NaCas (approximately 58 mmol/100 g) (J. Liu, Klebach, Visser, & Hofman, 2019), and the saturation point of NaCas succinylation is 4 mol SA/1 mol lysine (Shilpashree, Arora, Chawla, et al., 2015) which is equivalent to 23.2 g SA/100 g NaCas. Based on this, we performed the succinylation on the SA/NaCas at mass ratio of 5, 10, 15, and 20 *w*/w%, and found that all the modifications achieved high protein recovery yield (>80%, data not shown). The degree of succinylation (DS) increased significantly then gradually reached a plateau at ∼95% (**Fig. S2A**). The zeta-potential also showed a sharp decrease from less than −5 mV to −17.73 mV when SA to NaCas ratio increased from 0 to 15 w/w% (**Fig. S2B**). Therefore, to get a higher DS while minimizing the addition of SA, a 15% mass ratio of SA was chosen for further research, and S.NaCas hereafter refers to the S.NaCas prepared from the 15 w/w% addition of SA.

To confirm the success of succinylation, the FT-IR spectra for SA, and both native and modified NaCas were collected (**Fig. S3**). After modification, the adsorption band at 1740 cm^−1^ and the two bands at 1217, 1139 cm^−1^ were observed for S.NaCas, corresponding to the C=O and C—O stretching of the carboxyl group (Kamjornsupamitr, Hunt, & Supanchaiyamat, 2018; Ribeiro-Viana, Faria-Tischer, & Tischer, 2016). These bands confirm that a covalent bond formed between succinic acid and an amino group on the protein backbone. The band at 1367 cm^−1^ was from the stretching and bending vibration of -CH_2_, belonging to the succinic group (-CH_2_-COO^−^) (Kamjornsupamitr et al., 2018). At the same time, the C=O stretching bands at 1783 (asymmetric) and 1862 cm^−1^ (symmetric) disappeared which is indicative of the ring-opening reaction of SA in aqueous solution (Shilpashree, Arora, Sharma, & Singh, 2015).

To further characterize the S.NaCas, the particle size at pH 7.0 and zeta-potential at pH 2 through 10 of native and S.NaCas were analyzed (**Fig. S4**). S.NaCas showed an increase in particle size compared with NaCas, which can be explained by the introduction of the succinyl group onto the protein structure, as well as to a slight amount of protein aggregation during modification. The particle size distribution (PSD) of native and S.NaCas both showed a narrow single peak with a polydispersity index (PDI) < 0.3, which indicated the modification did not affect the overall size distribution of the protein. The zeta-potential within the pH range showed the net charge change during modification, confirming the succinylation induced more negative charges to the protein and led to a significant decrease of zeta-potential of S.NaCas above the pI.

### 3.2 SEI analysis between LF and S.NaCas

**Fig. 1:**
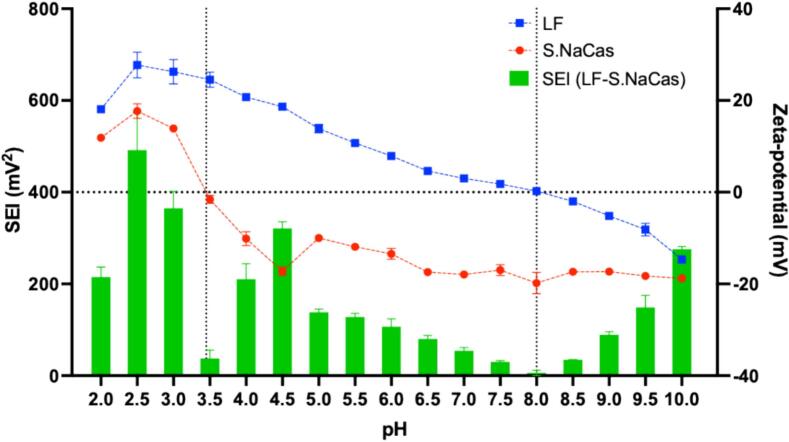
The zeta-potential of 2 mg mL^−1^ LF and S.NaCas and their strength of electrostatic interaction (SEI) at pH 2–10.

Using zeta-potential measurements the pI of LF was found at pH 8 while for S.NaCas it was 3.5, this indicated that these two proteins could form electrostatic complexes between pH 3.5 and 8.0 (Fig. 1). According to the strength of electrostatic interaction (SEI) measurement, pH 4.5 showed the largest SEI, so pH levels of 4.5 and 5.0 were chosen to form LF-S.NaCas complex. Although the SEI was high at pH 4.0, because this value was close to the pI the solubility of S.NaCas was poor and therefore, it was not chosen.

### 3.3 Formation of LF-S.NaCas complex

LF-S.NaCas binary complexes were formed at pH 4.5 and 5.0 under different LF to S.NaCas mass ratios using the pH first method (**Fig. S5**). The optical photos showed that insoluble complexes were formed at certain ratios under both pH levels (**Fig. S5A**). For electrostatic complexation, the optimal condition is the ratio at which the sample has the highest turbidity and yield. This is also the condition where the net charges are neutralized and the electrical double layer (EDL) stabilizing the solution is weakened or destroyed, therefore, the intermolecular forces of attraction between the biopolymers increase and an insoluble complex is formed and precipitated from solution. For LF to S.NaCas mass ratios of 1:2 at pH 4.5 and 2:1 at pH 5.0, the complexes showed the highest turbidity (**Fig. S5A, B**) and the zeta-potential was also close to zero (**Fig. S5C**). The optimal LF to S.NaCas mass ratio changed from 1:2 at pH 4.5 to 2:1 at pH 5.0 because as the pH increased, the positive charge of LF decreased and the negative charge of S.NaCas increased. So, more LF was needed to provide enough positive charge to achieve electrostatic neutralization.

Although the LF-S.NaCas complex can be formed in very high yield (∼ 90%) at both pH levels, S.NaCas itself was already a little turbid and formed aggregates itself at pH 4.5 (**Fig. S4A**), which had an adverse impact on complex formation. Moreover, we intended to add iron(II) to form the LF-S.NaCas-Fe ternary complex, and as the FeSO_4_ solution is acidic (1 M FeSO_4_ stock solution pH < 4.0), its addition would further decrease the pH and cause more S.NaCas to self-aggregate thus compromising the complexation. Therefore, pH 5.0 was chosen as the optimal ratio for iron co-encapsulation.

### 3.4 Formation and characterization of LF-S.NaCas-Fe complex

#### 3.4.1 Formation of LF-S.NaCas-Fe complex and effect of Fe concentration on complex formation

**Fig. 2:**
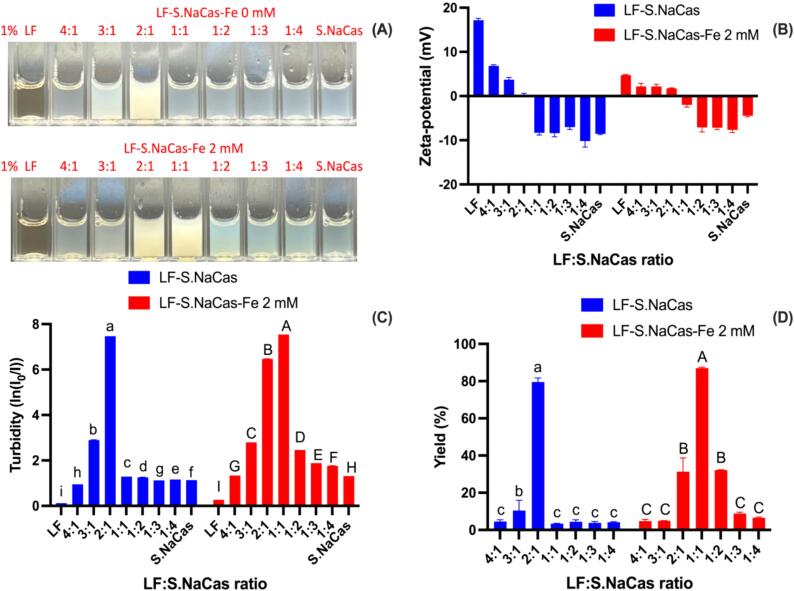
Optical image (A), zeta-potential (B), turbidity (C) and complex yield (D) of LF-S.NaCas and LF-S.NaCas-Fe 2 mM complex under different mixing ratios. All complexes were prepared at pH 5.0, protein concentration 10 mg mL^−1^. The different letters in each ratio indicate a significant difference of the turbidity and yield based on Student's *t-*test (*P* < 0.05).

Using the optimal conditions of pH 5.0 and LF to S.NaCas ratio of 2:1 for the binary complex, FeSO_4_ was then added to form the LF-S.NaCas-Fe ternary complex (Fig. 2). The Fe^2+^ concentration was fixed at 2 mM in complex mixtures with different protein ratios to form ternary complexes. The ternary complexes showed the same trend as the binary complex where turbidity (Fig. 2A-B) increased and then gradually reached the maximum when the zeta-potential (Fig. 2C) approached zero, then decreased again, consistent with the yield trend (Fig. 2D). The optimal LF to S.NaCas ratio needed to be reduced from 2:1 to 1:1 after adding iron since the positively charged Fe^2+^ competed with LF to bind with the negatively charged S.NaCas. This led to a decrease of LF content, which helped to confirm that complex formation was driven by electrostatic interactions.

**Fig. 3:**
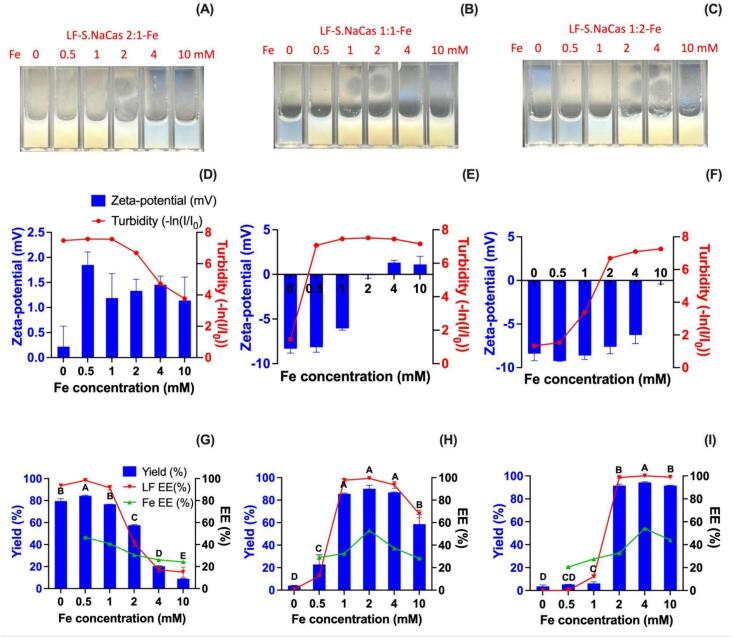
The effect of Fe concentration on the LF-S.NaCas-Fe ternary complex formation. Optical image (A-C), zeta-potential, turbidity (D—F), and complex yield, LF and Fe encapsulation efficiency (%) (G-I) of complex under different protein mixing ratios and iron concentrations. (LF to S.NaCas ratios: A, D, G 2:1; B, E, H 1:1; C, F, I 1:2) All complexes were prepared at pH 5.0, protein concentration 10 mg mL^−1^. The different letters in each ratio indicate a significant difference of the yield based on Student's t-test (P < 0.05).

In order to further explore the effect of iron concentration on complex formation, we chose different LF to S.NaCas ratios 2:1, 1:1, and 1:2, and changed the Fe^2+^ concentration from 0 to 10 mM to study their complex formation condition (Fig. 3).

**Fig. 4:**
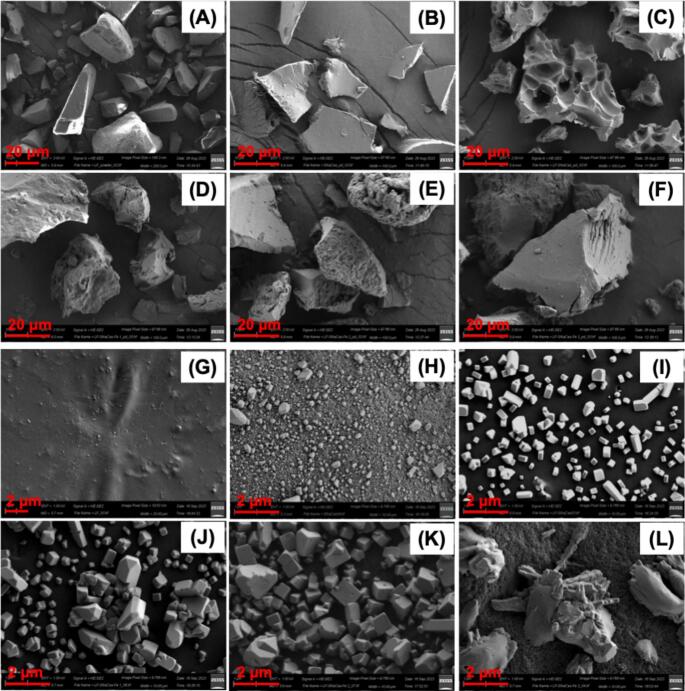
SEM image of ***freeze-dried powder*** (A-F) and ***protein solution/complex mixture*** (G-L) of LF (A, G), S.NaCas (B, H), LF-S.NaCas 2:1 complex (C, I), LF-S.NaCas 2:1-Fe 1 mM (D, J), LF-S.NaCas 1:1-Fe 2 mM (E, K) and LF-S.NaCas 1:2-Fe 4 mM (F, L) complex. A-G: scale bar 20 μm; G-L: scale bar 2 μm.

At pH 5.0, LF-S.NaCas binary complex reached the net charge neutralization at the ratio 2:1 and showed the highest turbidity and yield (Fig. 3A, D, G). With the addition of Fe^2+^, due to the.

introduction of positive charges, the overall electrostatic neutralization state could no longer be maintained at this ratio, the zeta-potential increased, and the formation of complexes decreased, showing lower yield. The shift of the optimal ratio was also observed in the other two ratios, with the LF-S.NaCas-Fe complexes reaching the highest turbidity, highest yield and near neutral zeta-potential at 2 mM Fe^2+^ for a 1:1 ratio of LF to S.NaCas (Fig. 3B, E, H) and 4 mM for 1:2 ratio of LF to S.NaCas (Fig. 3C, F, I).

The competition between binding sites by Fe^2+^ and LF provided further evidence that the ternary complexes were formed by electrostatic interactions. As the Fe^2+^ concentration increased, the optimal conditions for complex formation gradually shifted to less LF.

Meanwhile, different from LF-S.NaCas complex only forming at one specific ratio at pH 5.0 (Fig. 2A), the ternary complexes with high turbidity and yield could be formed at more than one ratios (Fig. 3A-C), which suggested that Fe promoted complex formation. But the mixture of Fe^2+^ and S.NaCas showed no obvious protein precipitation (**Fig. S6**) (Shilpashree, Arora, Sharma, & Singh, 2015), indicating that the yield increase was related to the ternary interaction. We postulated that the increase was because Fe^2+^ chelated with the proteins and served as a cross-linker in complex formation to strengthen the structure.

#### 3.4.2 Encapsulation efficiency (EE) and loading capacity (LC) of LF and Fe

The encapsulation of the LF and Fe^2+^ in the formed complex under different protein mixing ratios and Fe concentrations was also investigated (Fig. 3G-I).

For LF to S.NaCas mass ratio 2:1, the binary complex had a very high LF EE (>90%, Fig. 3G). After adding Fe^2+^, the LF EE first increased slightly and then decreased, showing a maximum at an Fe concentration of 0.5 mM, which was consistent with the highest yield at this ratio. With such a high complex yield (∼ 80%), most of the LF formed the insoluble complex and was encapsulated. As more Fe was added, the complex formation was compromised, so LF EE decreased gradually. For the other two mass ratios (1:1 and 1:2), the LF EE was also consistent with the yield trend, increasing and reaching almost 100% at an Fe^2+^ concentration of 2 mM at a LF:S.NaCas mass ratio of 1:1 and 4 mM for LF:S.NaCas mass ratio of 1:2 (Fig. 3H, I) before decreasing.

**Fig. 5:**
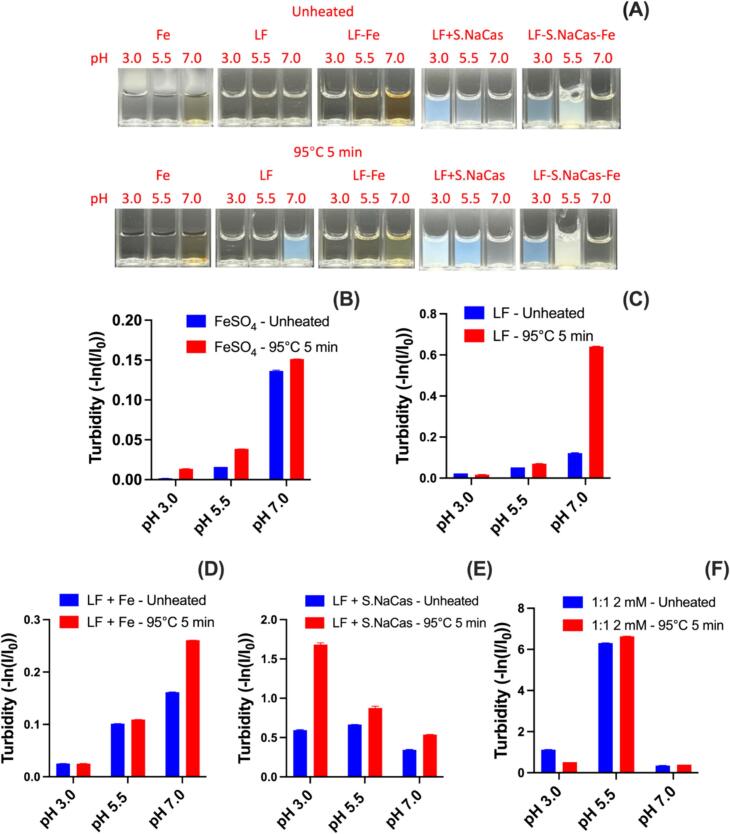
Optical images (A) and turbidity of FeSO_4_ (B), LF (C), LF + Fe^2+^ mixture (D), LF + S.NaCas 1:1 direct mixture (E) and redispersed LF-S.NaCas 1:1-Fe 2 mM complex (F) before and after thermal treatment under different pH levels.

The optimal EE for Fe^2+^ also coincided with the highest complex yield. At LF:S.NaCas mass ratio of 2:1, the Fe^2+^ EE reached a maximum at 0.5 mM Fe^2+^, and then decreased as the Fe^2+^ concentration increased (Fig. 3G). For a fixed protein concentration and ratio, the bonded iron amount was the same, so the more iron that was added decreased Fe^2+^ EE. The encapsulation of iron relied not only on binding to the protein, but also on the formation and precipitation of the LF-S.NaCas-Fe complex. Therefore, more complex formation promoted the co-precipitation of Fe^2+^ with the complex, which means that the Fe^2+^ EE reaches a maximum at the same time as the highest complex yield. For both the 1:1 and 1:2 LF:S.NaCas mass ratios, initially the Fe^2+^ EE increased, and the peak Fe^2+^ EE corresponded to the complex with the highest yield. With the Fe^2+^ concentration further increasing (from 2 to 4 mM at 1:1 ratio and 4 to10 mM at 1:2 ratio), the protein ratio and complex yield were similar while the Fe^2+^ EE decreased (Fig. 5H, I). This suggested that at lower Fe^2+^ concentrations, the increase of complex yield was the dominant factor facilitating Fe encapsulation. After the yield reached its maximum, the redundant Fe could no longer precipitate with the proteins and therefore, the EE of the Fe^2+^ decreased.

Based on the above results, the complexes with the highest LF and Fe^2+^ EE at each protein mixing ratio were selected as the optimal samples for further investigation. Their complex yield, EE, and LC are listed in Table 1. These three samples showed high yield (75% ∼ 90%), Fe^2+^ EE (∼ 50%) and LF EE (> 90%). Their iron loading reached between 2 and 12 mg/100 mg of the complex and varied at concentrations between 1 and 4 mM Fe^2+^. The LF LC corresponded to the initial LF mass ratio in each sample, with 691 mg LF/g complex at an LF to S.NaCas mass ratio of 2:1, 498 mg g^−1^ at an LF to S.NaCas mass ratio of 1:1, and slightly lower, 251 mg g^−1^ at an LF to S.NaCas mass ratio of 1:2.

**Table 1:** Optimized LF-S.NaCas-Fe complex formulas and the corresponding complex yield, encapsulation efficiency (EE), and loading capacity (LC) of Fe and LF.

Sample LF-S.NaCas-Fe	Complex yield (%)	Fe^2+^ EE (%)	LF EE (%)	Fe^2+^ LC (mg g^−1^)	LF LC (mg g^−1^)
LF-S.NaCas 2:1-Fe 1 mM	76.65 ± 0.08	46.88 ± 0.35	91.63 ± 2.17	2.46 ± 0.07	691.16 ± 1.66
LF-S.NaCas 1:1-Fe 2 mM	84.65 ± 0.06	53.56 ± 0.00	100 ⁎	6.89 ± 0.02	497.63 ± 1.76
LF-S.NaCas 1:2-Fe 4 mM	89.76 ± 0.67	54.12 ± 0.18	100 ⁎	12.06 ± 0.27	251.10 ± 5.08

⁎ LF not detected in the supernatant.

#### 3.4.3 Microstructure and morphology analysis of LF-S.NaCas-Fe complex

To investigate the microstructure and morphology of the complexes, SEM images of both freeze-dried powders and vacuum-dried complex mixtures were collected (Fig. 4 **and S7**). Then EDX spectra and elementary analysis were conducted based on the complex mixture samples to confirm the encapsulation of iron(II) in the complex (**Fig. S8 and Table S1**).

Powder samples of both LF and S.NaCas (Fig. 4A, B) showed relatively smooth surfaces, which was consistent with literature (Mittal et al., 2016). After the LF-S.NaCas binary complex was formed and freeze-dried, the particles exhibited a rough structure (Fig. 4C), and the holes on the surface could be due to the cavities left by the ice crystals during the freeze-drying process. However, after adding iron (Fig. 4D-F), this rough structure disappeared in the ternary complexes, and instead, the particle surface gradually became smoother as the iron concentration increased. This observation is likely a result of iron(II) acting as a cross-linker in the complex formation. This is not entirely unexpected as Fe^2+^ ions have been proven to form strong coordination bonds with oxygen atoms of phosphoseryl, aspartyl, and glutamyl residues of S.NaCas (Sun et al., 2020; Tian et al., 2021). Additionally, we found that adding iron(II) to solutions of LF caused the intrinsic fluorescence of LF to quench, which indicated that there was also interaction between LF and iron(II). Therefore, when iron(II) acted as a cross-linker and interacted with the two proteins, the complex structure became more compact, the rough structure disappeared and a smooth surface gradually formed.

To investigate if the centrifugation and freeze-drying process would impact the particle shapes and surface morphologies of complexes, SEM images of the protein solution/complex mixture samples after vacuum drying were also captured. The images reflected that the particle size was about 60 and 400 nm for 10 mg mL^−1^ LF and S.NaCas, respectively (Fig. 4G, H). The S.NaCas size was consistent with the particle size measured by direct light scattering (DLS) (**Fig. S2C**), though there were some large particles, which may be due to the protein aggregation during protein modification.

After forming the complex, the LF-S.NaCas sample showed a very regular cubic morphology (Fig. 4I), and the particle size was slightly larger than that of S.NaCas, between 500 nm to 1 μm. This cube-shape has also been reported in a casein-containing complex recently (Alrosan et al., 2023). Alrosan et al. claimed that the surface morphological alteration was related to protein surface hydrophobic interactions, but the detailed mechanism has not yet been revealed. In general, complexes either form spherical coacervate droplets under specific conditions (pH, concentration, ionic strength, protein ratio) or amorphous complex aggregations (Gigli, 2016). Why such regular cubic nanoparticles would form between LF and S.NaCas requires further study. However, the addition of iron also caused obvious changes in the structure of the complex in the vacuum-dried samples, which was manifested by gradually increasing particle size and irregular surface morphology. In the complexes formed with 1 and 2 mM Fe^2+^ (Fig. 4J, K), some irregular particle aggregation was observed, with particle sizes ∼1 μm, while the surface of the sample of the complex formed with 4 mM Fe^2+^ (Fig. 4L) was composed of aggregates of large particles (∼3 μm) with amorphous morphology. The surface morphology and particle size change also supports the cross-linking effect of Fe^2+^.

In order to confirm the loading of Fe^2+^, we conducted the element analysis (EDX) based on SEM images (**Fig. S8 and Table S1**). Due to the carbon coating, the carbon content dominated in all samples, as evidenced by elemental analysis of the background captured in the empty field without particles (**Fig. S8A**). While nitrogen only came from protein samples (showing negative values in background, **Table S1**), it can be used as an indication element for protein complexes and showed a high content in all LF-S.NaCas/-Fe complexes (11%–17%). In the binary complexes, there was little iron(III) from LF, accounting for 0.01% of all atoms. After adding iron(II), higher iron contents were detected in three ternary complexes, ranging from 0.17% to 0.44% atomically, proving the successful loading of Fe^2+^. The high sodium content in the samples (accounting for 2% to 8.65%) mainly came from the S.NaCas, a sodium salt itself, and also from the complex formation process, in which the pH is adjusted by NaOH and HCl which introduce a certain amount of Na^+^ and Cl^−^ ions, thus resulting in high levels of these two elements.

### 3.5 Thermal study of LF-S.NaCas-Fe complex

#### 3.5.1 Effect of pH on the thermal stability of components and complexes

Through electrostatic complexation of LF with thermally stable NaCas and the further cross-linking of the two biopolymers by Fe, we postulate that the ternary complex system will improve the thermal stability of LF and iron solubility. To investigate the thermal behavior of the complexes in different food matrices, such as beverages, flour, and milk, during food processing and sterilization, the pH levels 3.0, 5.5, and 7.0 were chosen as the model of acid, weak acid, and neutral food conditions. To better mimic the industrial processing, an aggressive heating condition, 95 °C for 5 min, was applied to test the thermal stability of LF, FeSO_4_ and S.NaCas, separately, the binary mixture of LF and Fe^2+^, the direct mixture of LF and S.NaCas, as well as the LF-S.NaCas-Fe complex (LF-S.NaCas 1:1-Fe 2 mM was chosen as the representative).

We first confirmed the thermal stability of modified S.NaCas. The optical image and turbidity (**Fig. S9A**) indicated that S.NaCas (2 mg mL^−1^) was thermally stable in both weakly acidic (pH 5.5) and neutral (pH 7.0) environments, having a clear solution before and after heating, with almost no change in turbidity (S.NaCas had poor solubility at pH 3.0, which is too close to its pI). Particle size and zeta-potential (**Fig. S9B—D**) also remained stable at pH 5.5 and 7.0 during thermal treatment of NaCas. This high thermal stability of NaCas was partially because caseinate lacks a spatial protein structure and instead has a randomly coiled structure (Petukhov, Kil, Kuramitsu, & Lanzov, 1997). Since α-helix and β-sheet, stabilized by hydrogen bonds, are the vital part of protein secondary structure and heating causes protein unfolding and breakage of hydrogen bonds that leads to a decrease in the helix structure which then affects the protein functions (Corrêa & Ramos, 2009; Greenfield, 2004). Therefore, the random-coiled NaCas is quite stable to thermal treatment as confirmed by circular dichroism (CD) spectra (**Fig. S7E**) that showed the succinylation did not change the protein structure. There was a small peak for α-helix at 190 nm (∼ 5%, **Table S2**), but the larger negative band at ∼200 nm corresponded to the random coil structure (>30%) (Lin et al., 2022). During thermal treatment, there was almost no change in the CD spectra of S.NaCas at both pH 5.5 and 7.0, also proving that S.NaCas was still thermal stable and potential to protect LF from thermal denaturation and aggregation.

At pH 3.0, FeSO_4_ solution (0.4 mM) was clear both before and after heating (Fig. 5A**&B**), presenting good thermal stability in the acidic environment. Zeta-potential also remained stable during heating (**Fig. S10A**), indicating stable solubility and ionization at pH 3.0. At pH 5.5, the solution was still clear before heating, but visible yellow precipitate formed after heating (Fig. 5A). However at pH 7.0, the oxidative precipitation was observed in unheated samples and increased noticeably with heating, with >90% of iron(II) oxidized to iron(III) even before heating. Meanwhile, the zeta-potential of FeSO_4_ solution decreased to nearly zero after heating at pH 5.5 and 7.0 (**Fig. S10A**), potentially related to the formation of insoluble hydroxides or oxides, which led to lower iron bioavailability in the human body. Overall, the free iron(II) was thermally stable at low pH, and the stability decreased with pH increasing. At neutral pH, even unheated samples were not stable. Therefore, it is more important to address the iron solubility problem under neutral pH.

LF also showed good thermal stability in the acidic environment, which is consistent with previous reports (Goulding, O'Regan, Bovetto, O'Brien, & O'Mahony, 2021). The LF solution (2 mg mL^−1^) remained clear during heating at pH 3.0 and 5.5, with little change in turbidity (Fig. 5A, C) and particle size (**Fig. S11**). However, the heated LF at neutral pH showed an obvious increase in turbidity and particle size, indicating protein aggregation. The LF and Fe^2+^ mixture (adding FeSO_4_ stock solution direct to LF solution without adjusting the pH) showed similar thermal stability to the LF alone, showing no obvious visual changes after heating at pH 5.5 and below (**Fig. S12**). Their thermal stability under acidic condition was confirmed by the CD spectra and secondary structure analysis (**Fig. S11, S12D, Table S3, 4**). There was no significant α-helix structure decrease (decreasing by about 10% in the LF solution and almost no change in LF and Fe mixture). However, at pH 7.0, almost half of the α-helix structure was destroyed in LF during the heating process and replaced by an unordered structure, reflected by significant decrease of positive peak at 190 nm and stronger negative signal band at 200 nm, which was also observed in LF and Fe^2+^ mixture. The decrease in thermal stability of LF in neutral environments was due to the decrease in protein surface charge as the pH approached LF's pI so there was not enough electrostatic repulsion to maintain protein particles in the aqueous solution. When heated, as the hydrophobicity of the protein increased, and the hydrogen bonds weakened, LF aggregated and precipitated (Li & Zhao, 2017).

Solutions of LF and S.NaCas direct mixtures (DM) showed higher turbidity due to the low solubility of S.NaCas at pH 3.0 (Fig. 5A, E), and the turbidity increased after heating. However, the CD spectra (**Fig. S13D**) showed no obvious changes during heating, suggesting LF maintained thermal stability in the presence of S.NaCas. At pH 5.5, because it was close to the pH of complex formation, there was some electrostatic interaction between the two proteins, corresponding to a lower zeta-potential value and higher turbidity (Fig. 5E**, S13B**), and since heating might promote the formation of the complex, the turbidity further increased during thermal treatment. The overall CD signal weakened after heating at pH 5.5 (**Fig. S13D**) which might also be due to the low solubility of the protein. At pH 7.0, the DM of LF and S.NaCas had good solubility with no obvious change in turbidity, zeta-potential, particle size, PSD, or CD spectra (**Fig. S13**). These results indicated that DM already had some protective effect on LF.

As a representative of the ternary complex, LF-S.NaCas 1:1-Fe 2 mM complex presented good thermal stability at pH 7.0, showing clear solutions before and after heating with stable particle size, zeta-potential, and CD spectra after heating (Fig. 5A, F**, S14**). Because the other two pH levels were either close to the pI of S.NaCas or the pH that the complex was formed (net charge neutralized), the complex showed poor solubility at both pH 3.0 and 5.5. But considering both LF and Fe^2+^ were stable under acidic conditions, it could be implied that the complex should also be thermally stable at these pH levels. So generally, at pH 7.0, both LF and Fe^2+^ were not stable, while the LF-S.NaCas 1:1-Fe 2 mM complex showed good solubility and stability before and after heating at pH 7.0, which effectively solved the problems of iron(II) oxidation precipitation and thermal denaturation of LF.

#### 3.5.2 Thermal study of different redispersed complexes during thermal treatment in neutral pH

**Fig. 6:**
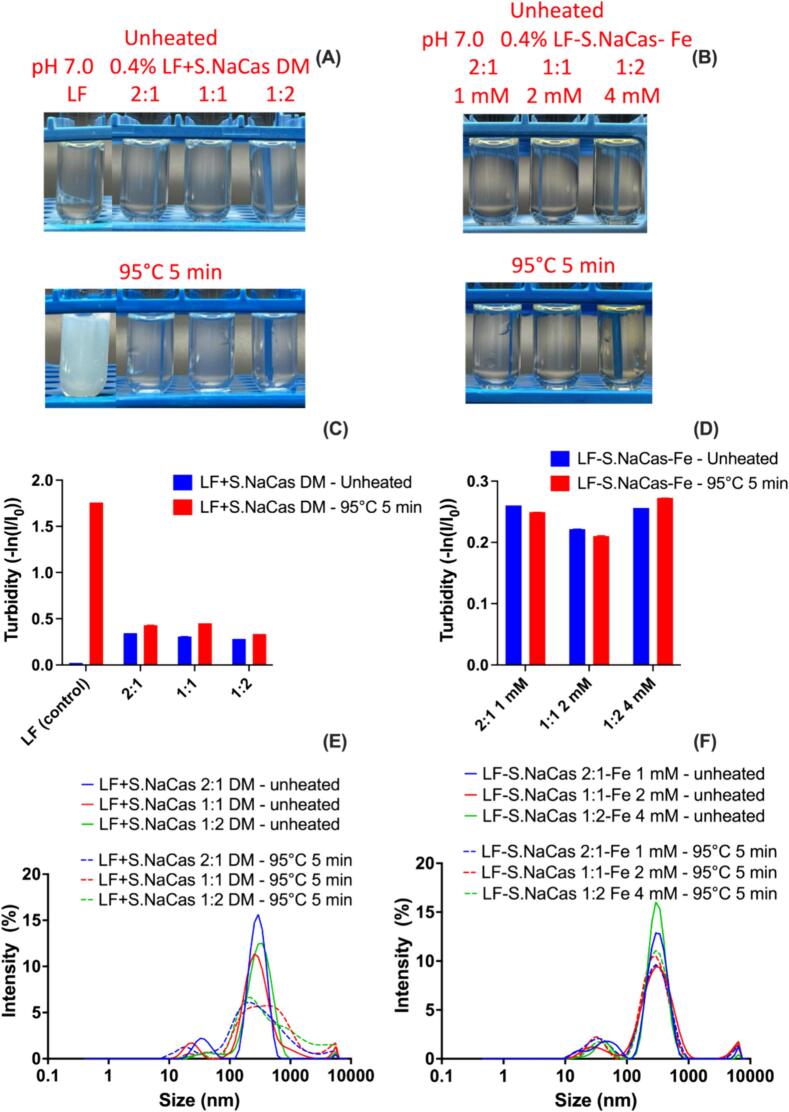
Optical images, turbidity (A, C) and size distribution (B, D) of LF + S.NaCas direct mixture and redispersed LF-S.NaCas-Fe complex before and after thermal treatment at pH 7.0.

After having an overall understanding of the thermal stability of the complex and each component at different pH levels, pH 7.0 was found to be more challenging for both LF and iron delivery, as indicated by the thermal denaturation of LF and the oxidative precipitation of iron. For most food matrices, the target pH is neutral, so our goal was to investigate the thermal stability of our complexes, especially those with higher LF concentrations at pH 7. For this purpose, we analyzed and compared the thermal stability of all three complexes and corresponding proportions of LF and S.NaCas DM (Fig. 6).

LF itself is thermally unstable and undergoes thermal denaturation and aggregation at pH 7.0, but simply mixing it with S.NaCas at 1:1 ratio improved its stability and prevented it from aggregating during thermal treatment. We found this improvement at all three ratios of LF to S.NaCas tested, 2:1, 1:1 and 1:2. Even at the higher LF to S.NaCas ratio 2:1, the DM solution remained clear after heating (Fig. 6A). The overlap of the CD spectra (**Fig. S15**) at each ratio also confirmed their thermal stability. From the secondary structure analysis, for each mixing ratio of DM, the α-helix and β-sheet structures of LF did not decrease noticeably during heating (**Table S6**). On the contrary, as the proportion of thermally stable S.NaCas increased, the α-helix of LF increased by 6% after heating. This can be explained from previous work (Li & Zhao, 2017) that reported LF and NaCas could form heat-induced complexes through not only electrostatic interactions, but also hydrophobic interactions and disulphide exchange interactions. Li & Zhao claim that the randomly coiled NaCas was adsorbed onto the surface of LF particles to form a core-shell or even micellar structure. Therefore, during the thermal treatment, LF might also interact with S.NaCas and form some complex through this mechanism. This could be also explained by the particle size change of the DM samples, which had a larger distribution at >1000 nm **(**Fig. 6E**)** with a higher intensity and wider peak width (**Fig. S16A**) after heating.

After the complex was formed, the thermal stability of LF was further strengthened compared with DM. All three complexes with different LF ratios and Fe concentrations remained clear solution before and after heating, without increase in turbidity or particle size (Fig. 6C **and S16B**). The PSD (Fig. 6F) maintained sharp and narrow peaks, indicating that the complex particles were still uniformly distributed after heating. For the CD results, although the spectra of all three complexes overlapped **(Fig. S15**), the sample with high LF content (LF:S.NaCas 2:1, Fe 1 mM), still went through a slight decrease of α-helix from 10.8% to 9% (**Table S6**), which was because the amount of S.NaCas was not sufficient to provide LF enough protection. When the S.NaCas ratio was increased to equal or above the LF amount, the sample no longer showed reduction in α-helix. Unlike the DM samples, the complexes showed no obvious increase in α-helix, this was likely due to the complexed protein no longer being capable of hydrophobic or disulphide interactions. Therefore, the complexes were able to maintain their structures, but could not produce a more ordered structure. In conclusion, at pH 7.0, all three complexes showed good solubility and thermal stability, without obvious LF aggregation or Fe precipitation.

#### 3.5.3 LF retention and Fe release after thermal treatment

**Fig. 7:**
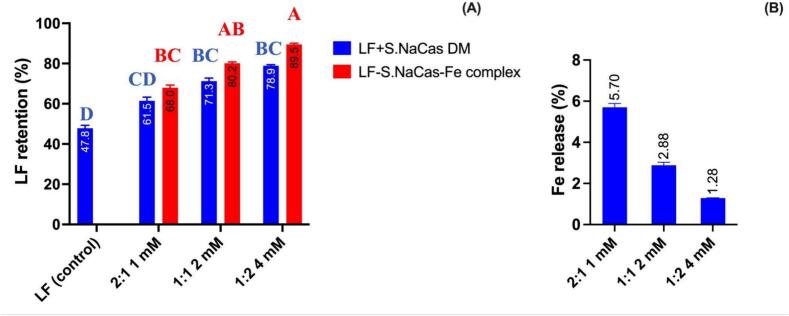
LF retention of the direct mixture of LF and S.NaCas, and redispersed LF-S.NaCas-Fe complexes (A) and Fe release of complexes (B) with different mixing ratios and Fe concentration during thermal treatment at pH 7.0. The different letters in the figure indicate a significant difference based on Students's *t-*test (*P* < 0.05).

To quantitatively analyze the thermal denaturation of LF, the LF retention percentage of DM and complex samples after heating was analyzed by HPLC (Fig. 7A).

For pure LF (2 mg mL^−1^), after heating at 95 °C for 5 min in neutral pH, there was <50% retention, showing poor thermal stability. Whereas, both DM and complex samples had better thermal stability and higher LF retention compared to pure LF, and this improvement increased with a higher S.NaCas ratio. In the DM samples, the LF retention was increased to 61.5% for the LF to S.NaCas mass ratio of 2:1 and 78.9% for the LF to S.NaCas mass ratio of 1:2. In LF-S.NaCas.Fe complexes, LF thermal stability further improved with 68.0 % retention of LF for a 2:1 ratio of LF to S.NaCas ratio of 2:1, 80.2% for a 1:1 ratio and 89.5% at a 1:2 ratio. This was because the more thermally stable S.NaCas helped stabilize LF, so in both DM and complex samples the LF showed higher retention in samples with higher S.NaCas ratio. The complexes possessed even higher LF retention compared with DM, proving the complexation process provided better protection for LF from thermal denaturation. The cross-linking of Fe^2+^ might be another reason that the complexes retained more LF, especially for those with higher Fe^2+^ concentration and a higher S.NaCas ratio.

Overall, LF-S.NaCas-Fe complexes showed enhanced thermal stability, and for all three complexes, the LF retention was significantly different (*p* < 0.05) compared with pure LF. As the S.NaCas ratio and Fe^2+^ concentration increased, the complex showed increased LF retention. After heating we found that almost 90% of the LF was protected from denaturation in the complex LF-S.NaCas 1:2-Fe 4 mM, whereas LF when heated by itself was found to be denatured by almost 50%. However, the higher S.NaCas content in this complex might compromise LF loading capacity despite showing better thermal stability. We found that even for the complexes with higher LF content, >68% of the LF was protected from denaturation which was still a significant improvement compared to unprotected LF.

Fe^2+^ release after thermal treatment was measured to evaluate the Fe^2+^ encapsulation strength, expressed as the percentage of iron released to the total iron (Fig. 7B). Low iron release was observed in all three complexes (below 10%), and at higher S.NaCas ratio, only about 1.5% of Fe^2+^ was released from the complex. This was due to the strong binding of Fe^2+^ to S.NaCas, as indicated by the indirect relationship between the decrease in Fe^2+^ release with an increase in S.NaCas concentration. Our complexes provide a food safe and efficient solution for Fe encapsulation which addresses the poor solubility of Fe^2+^ in neutral pH.

## 4 Conclusion

A simple but effective LF-Fe co-encapsulation and delivery system was established by taking advantage of the iron binding of S.NaCas and electrostatic interaction between LF and S.NaCas. Through the electrostatic interaction between LF and S.NaCas, we successfully prepared the LF-S.NaCas hetero-protein complex with high yield (> 90%), with an optimal complexation ratio at pH 5.0 of LF to S.NaCas of 2:1. On this basis, through the interaction between iron and the two proteins, the LF-S.NaCas-Fe ternary complex was prepared with different LF to S.NaCas ratios and Fe^2+^ concentrations, achieving a maximum iron loading of up to >12 mg g^−1^ complex. At the same time, Fe^2+^ served as a cross-linker by chelating with both proteins and thus strengthening the complex structure. The iron containing ternary complex exhibited excellent thermal stability and prevented the temperature sensitive LF from thermal denaturation in neutral pH. This delivery system can address the thermal denaturation and aggregation of LF as well as increasing the solubility of Fe^2+^ in a neutral environment and may potentially improve its bioavailability. We expect this thermally stable co-delivery system can eventually be applied in iron-fortified and high protein functional foods.

## CRediT authorship contribution statement

**Yunan Huang:** Writing – review & editing, Writing – original draft, Investigation, Formal analysis, Data curation. **Tiantian Lin:** Writing – review & editing, Validation, Methodology, Investigation, Conceptualization. **Younas Dadmohammadi:** Writing – review & editing, Supervision, Funding acquisition, Conceptualization. **Yanhong He:** Writing – review & editing, Investigation, Conceptualization. **Waritsara Khongkomolsakul:** Writing – review & editing, Investigation. **Claire Elizabeth Noack:** Writing – review & editing, Investigation. **Alireza Abbaspourrad:** Writing – review & editing, Supervision, Resources, Project administration, Funding acquisition.

## Declaration of competing interest

The authors declare that they have no known competing financial interests or personal relationships that could have appeared to influence the work reported in this paper.

## Data Availability

Data will be made available on request.

## Appendix A Supplementary data

Supplementary material Image 1
