# Supplementary material for: Lactoferrin thermal stabilization and iron(II) fortification through ternary complex fabrication with succinylated sodium caseinate
Source: Food Chem X. 2024 May 23;22:101498. doi: 10.1016/j.fochx.2024.101498 (PMC11190486; doi:10.1016/j.fochx.2024.101498)
Supplement: Supplementary file 1 — Supplementary material [file mmc1.docx]

Lactoferrin Thermal Stabilization and Iron(II) Fortification through Ternary Complex Fabrication with Succinylated Sodium Caseinate

Yunan Huang ^a^, Tiantian Lin ^a^, Younas Dadmohammadi ^a^, Yanhong He ^a^, Waritsara Khongkomolsakul ^a^, Claire Elizabeth Noack ^a^, and Alireza Abbaspourrad ^a, *^

^a^ Department of Food Science, College of Agriculture and Life Sciences, Cornell University, Ithaca, NY, USA


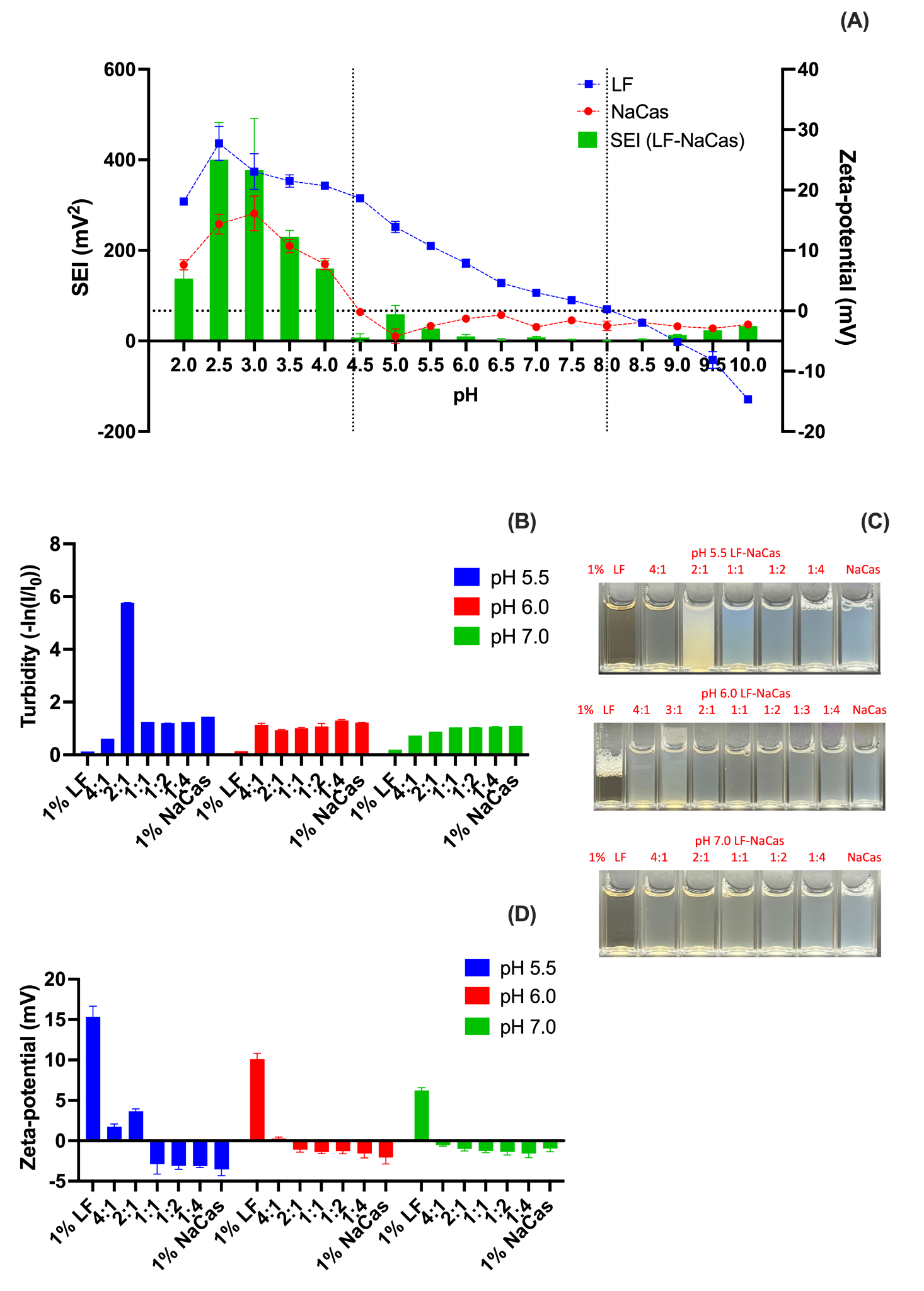


**Figure S1**. Formation of LF-NaCas complex: SEI result between LF and native NaCas (A); the Optical image (B), zeta-potential (C) and turbidity (D) of LF-NaCas complex under different pHs. All complexes are prepared with protein concentration 10 mg mL^–1^.


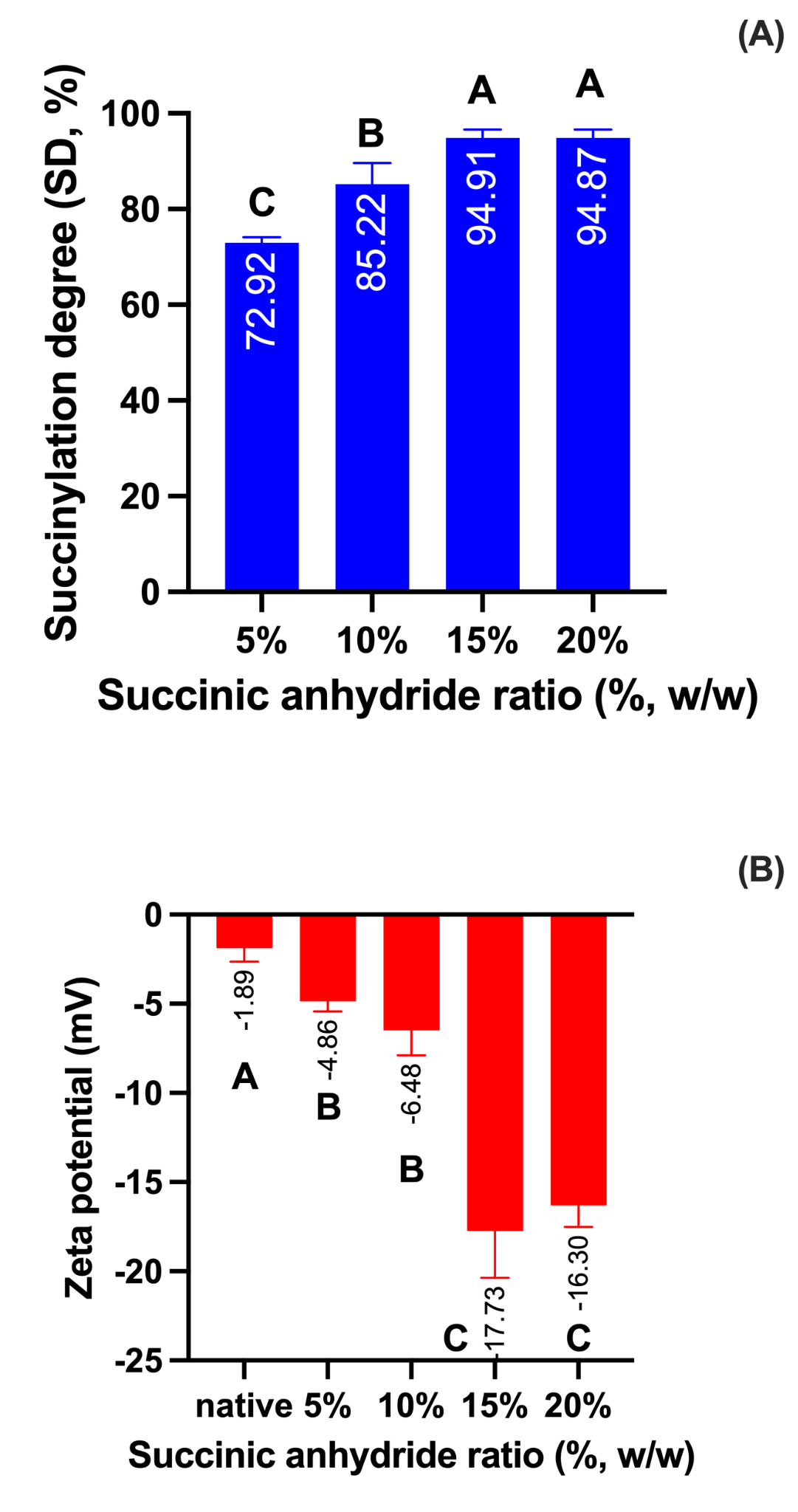


**Figure S2**. Succinylation degree of S.NaCas (A) and the zeta-potential of 1% native NaCas and S.NaCas with different succinic anhydride adding amount at pH 7.0 (B). Different letters on the columns indicate significant difference (p < 0.05).


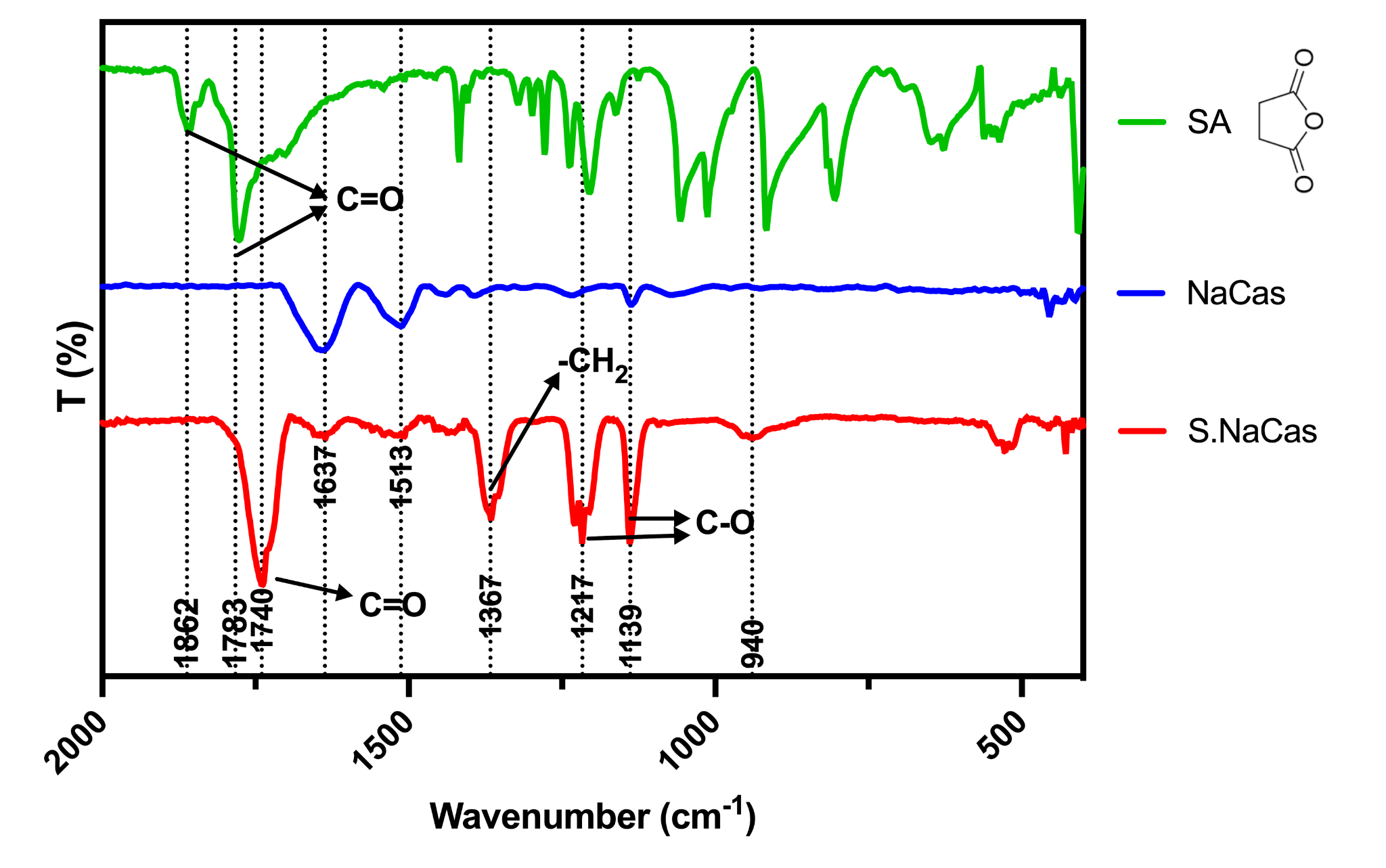


**Figure S3**. FT-IR spectra of SA, native and S.NaCas.


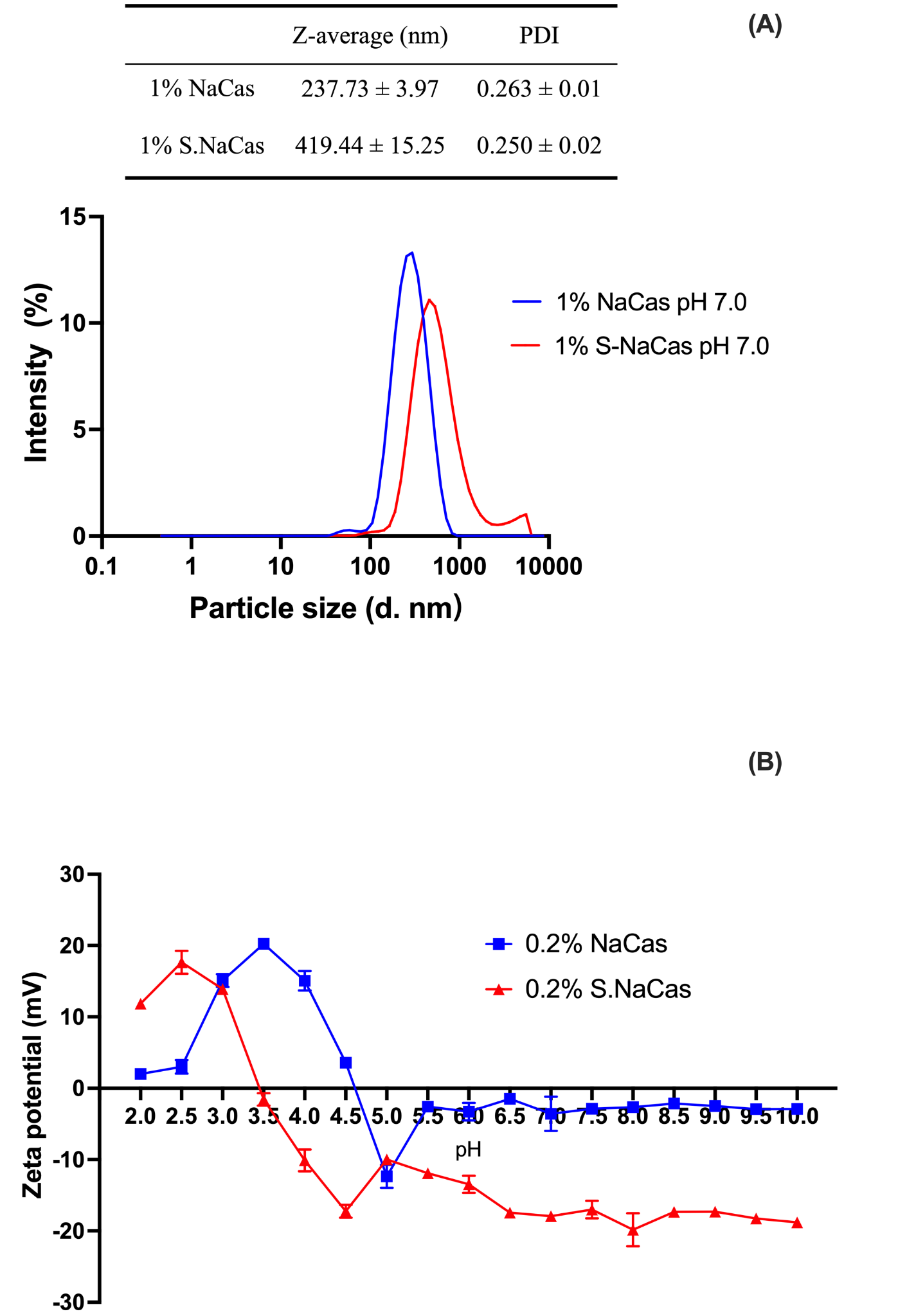


**Figure S4.** Characterizations of S.NaCas. Particle size and size distribution of 1% native and S.NaCas at pH 7.0 with 15% SA adding (A); zeta-potential within pH 2-10 of 0.2% native and S.NaCas with 15% SA adding (B).


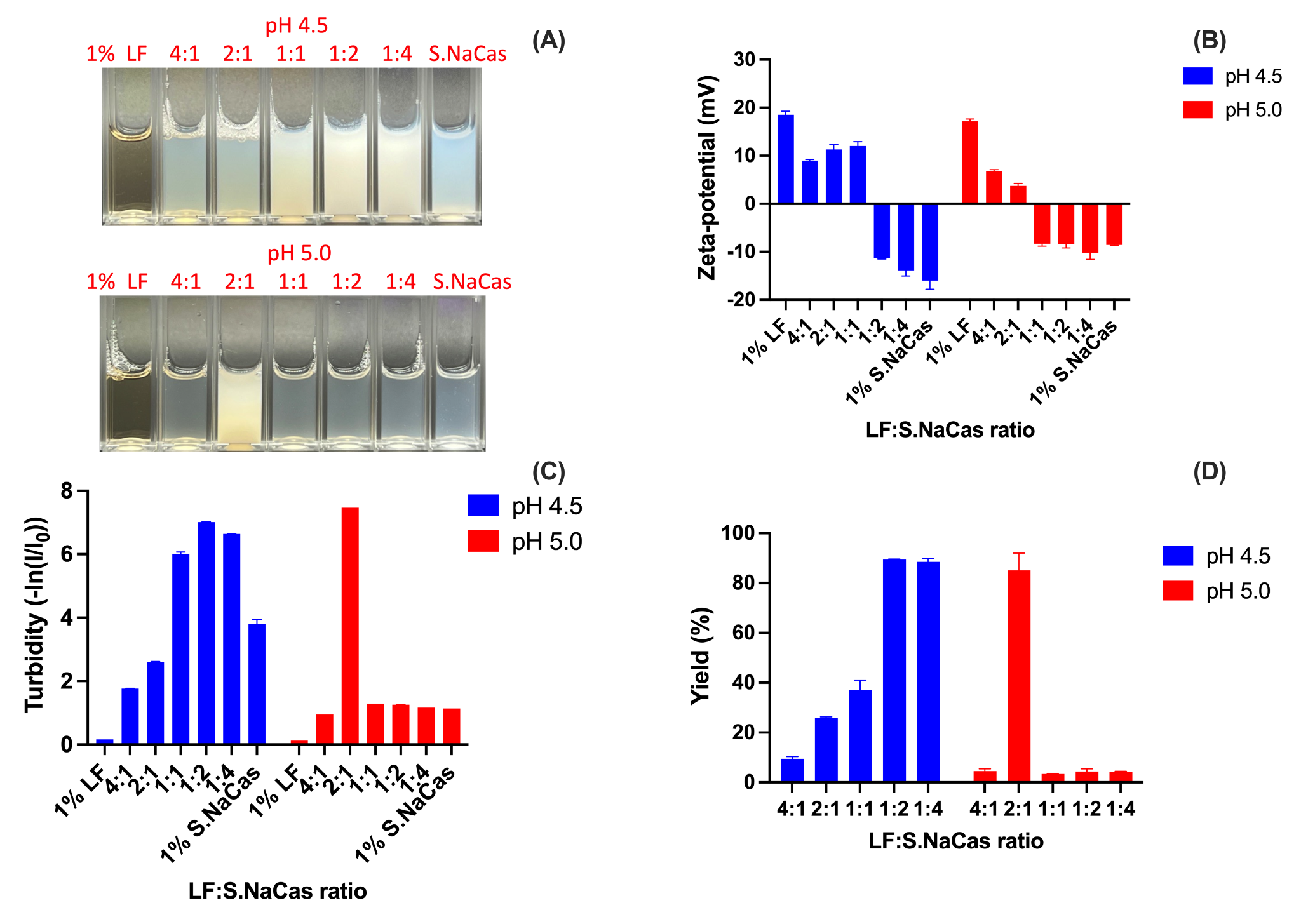


**Figure S5.** Optical image (A), turbidity (B), zeta-potential (C) and complex yield (D) of LF-S.NaCas complex under different pHs. All complexes are prepared with protein concentration 10 mg mL^–1^.


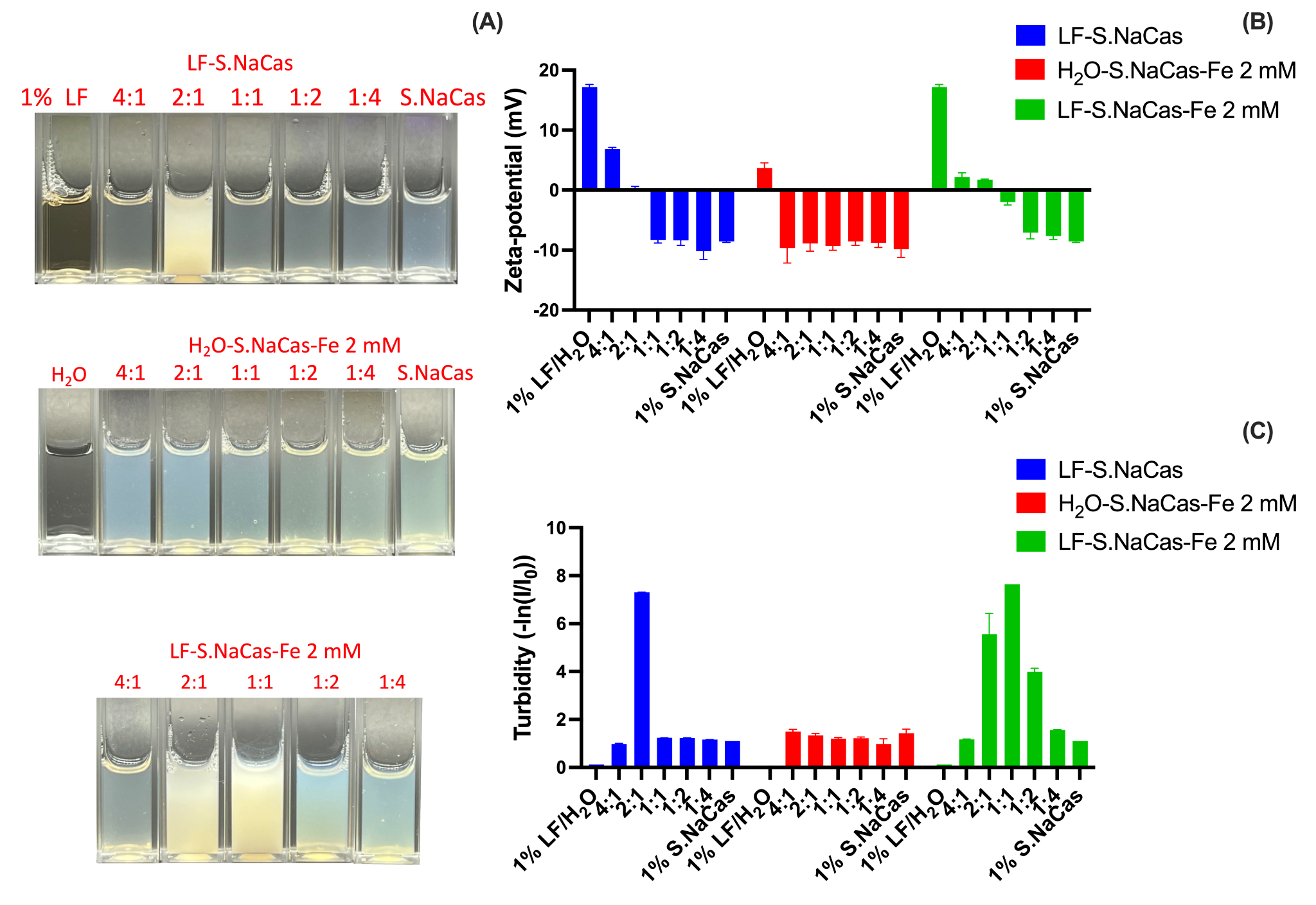


**Figure S6**. Comparison of the formation of the binary or ternary complex with S.NaCas-Fe mixture: the optical image (A), zeta-potential (B) and turbidity (C) of LF-NaCas-Fe complex and S.NaCas-Fe mixture. All complexes are prepared with protein concentration 10 mg mL^–1^.


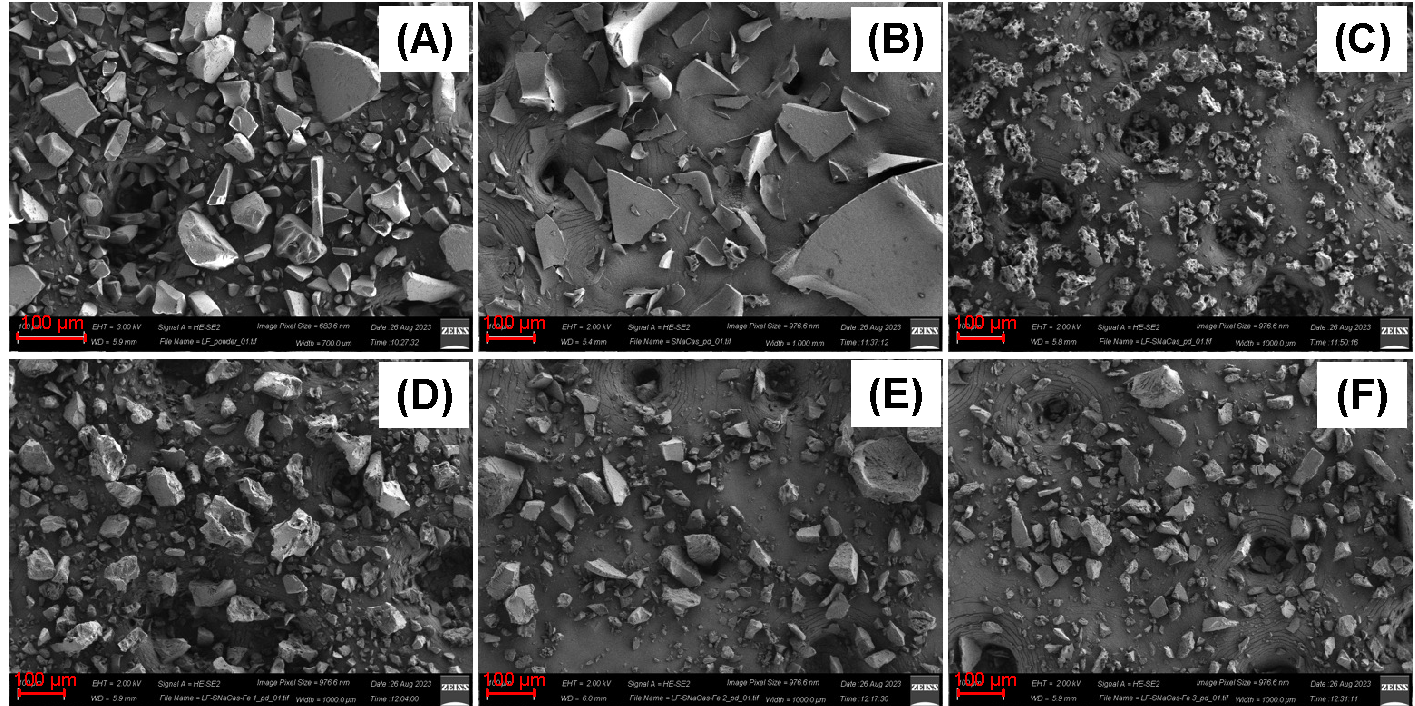


**Figure S7.** SEM image with larger view field of ***freeze-dried powder*** of LF (A), S.NaCas (B), LF-S.NaCas 2:1 complex (C), LF-S.NaCas 2:1-Fe 1 mM (D), LF-S.NaCas 1:1-Fe 2 mM (E) and LF-S.NaCas 1:2-Fe 4 mM (F) complex. Scale bar 100 μm.


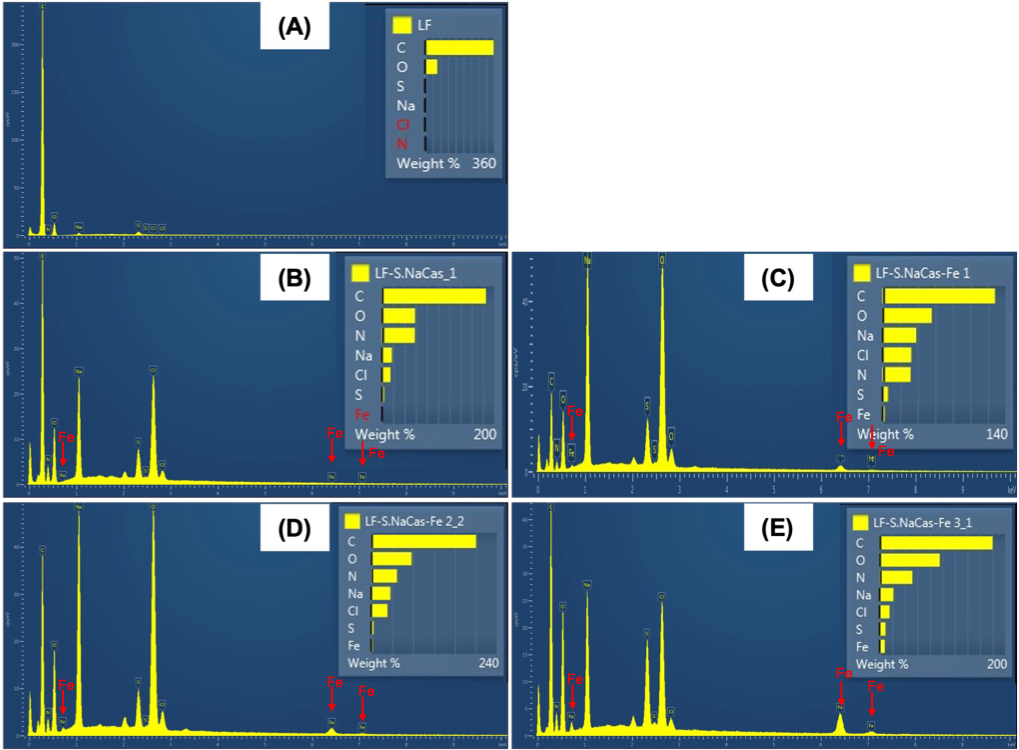


**Figure S8.** EDX spectra of stab pin background (A), LF-S.NaCas 2:1 complex (B), LF-S.NaCas 2:1-Fe 1 mM (C), LF-S.NaCas 1:1-Fe 2 mM (D), and LF-S.NaCas 1:2-Fe 4 mM (E) complex.

| Element | Atomic (%) | | | | |
| --- | --- | --- | --- | --- | --- |
|  | Background | LF-S.NaCas/-Fe complex | | | |
|  |  | 2:1 | 2:1 1 mM | 1:1 2 mM | 1:2 4 mM |
| C | 88.36 | 62.67 | 55.33 | 60.50 | 60.63 |
| N | -0.39 | 16.99 | 11.85 | 13.35 | 11.06 |
| O | 11.73 | 14.98 | 18.17 | 17.25 | 24.44 |
| Na | 0.15 | 3.14 | 8.65 | 5.32 | 2.00 |
| S | 0.14 | 0.47 | 0.99 | 0.51 | 0.89 |
| Cl | 0.00 | 1.73 | 4.79 | 2.89 | 0.54 |
| Fe | 0.00 | 0.01 | 0.23 | 0.17 | 0.44 |
| Total: | 100.00 | 100.00 | 100.00 | 100.00 | 100.00 |

**Table S1.** Elementary analysis of background and LF-S.NaCas/-Fe binary and ternary complexes based on the EDX analysis on Fig. S8.


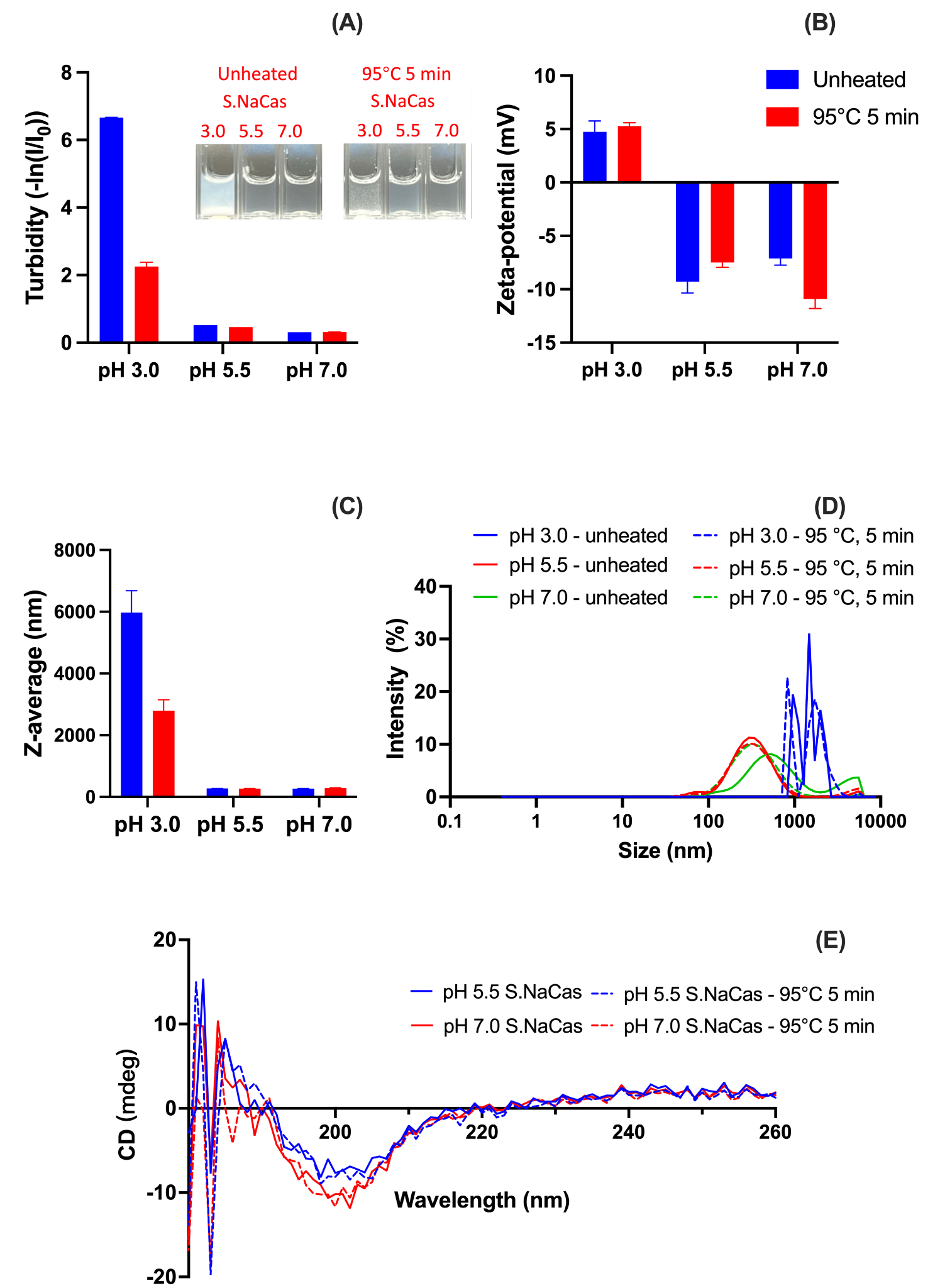


**Figure S9**. Optical image and turbidity (A), zeta-potential (B), particle size (C), size-distribution (D) and CD spectra (E) of 0.02 mg/mL S.NaCas under different pHs before and after thermal treatment. pH 3.0 gave no CD result because this pH was close to pI of S.NaCas, which had poor solubility.

**Table S2.** Simulated CD statistics of S.NaCas secondary structure percentage and structural change during heating under different pHs based on Fig. S5.

|  | | Helix (%) | Strand (%) | Turns (%) | Unordered (%) |
| --- | --- | --- | --- | --- | --- |
| pH 7.0 | Unheated | 6.05 ± 0.49 | 35.80 ± 1.27 | 21.75 ± 0.78 | 36.40 ± 0.00 |
|  | 95°C, 5 min | 5.75 ± 0.64 | 36.65 ± 0.92 | 21.65 ± 0.35 | 36.00 ± 0.00 |
|  | **Change (%)** | **-4.21** | **2.48** | **-0.37** | **-1.10** |
| pH 5.5 | Unheated | 5.70 ± 0.57 | 36.90 ± 0.71 | 21.45 ± 0.35 | 35.95 ± 0.49 |
|  | 95°C, 5 min | 5.85 ± 0.35 | 36.75 ± 0.92 | 22.25 ± 0.21 | 35.25 ± 0.35 |
|  | **Change (%)** | **2.83** | **-0.41** | **3.75** | **-1.94** |


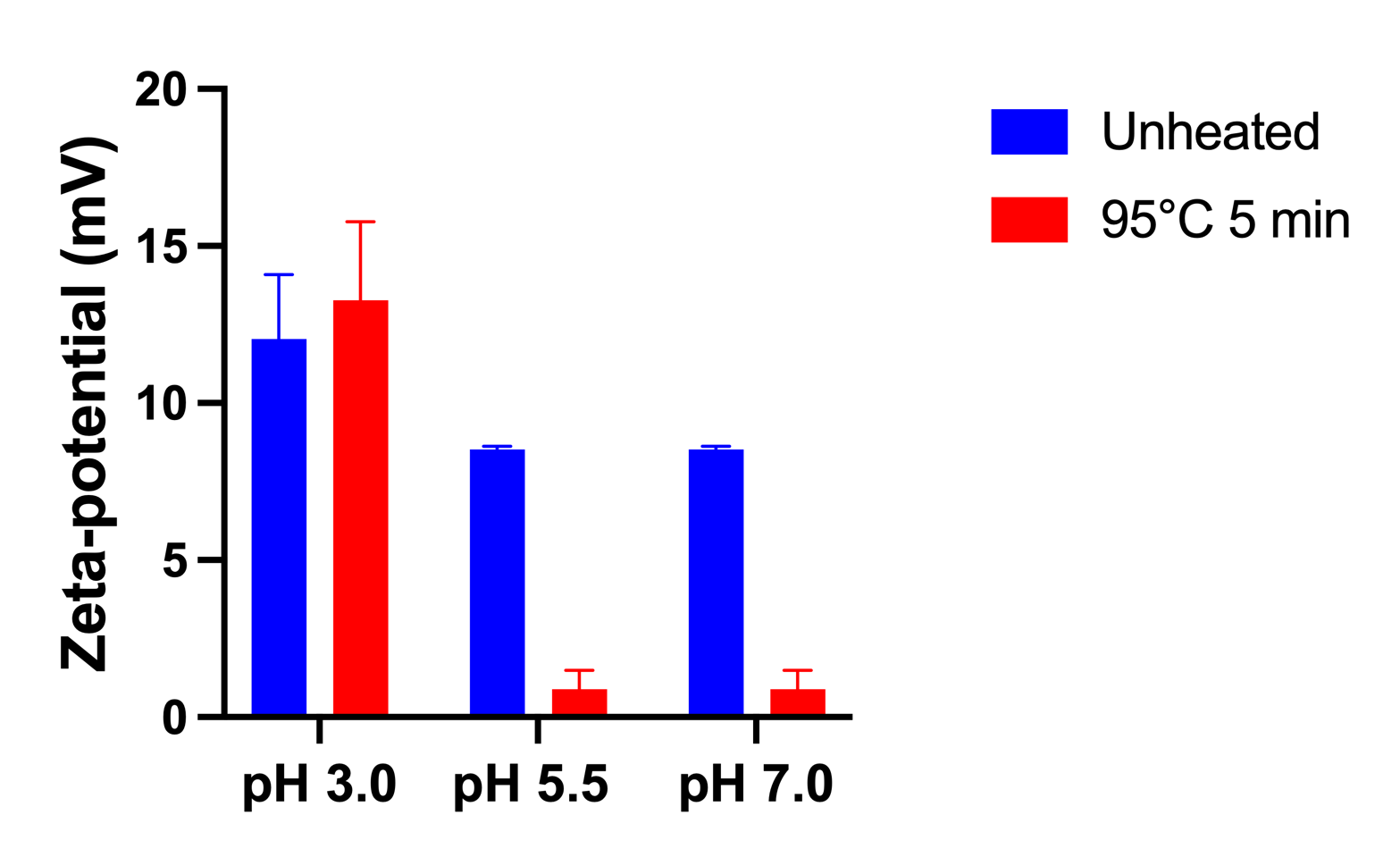


**Figure S10.** Thermal stability of FeSO_4_. Zeta potential of 0.4 mM FeSO_4_ during thermal treatment under different pH levels.


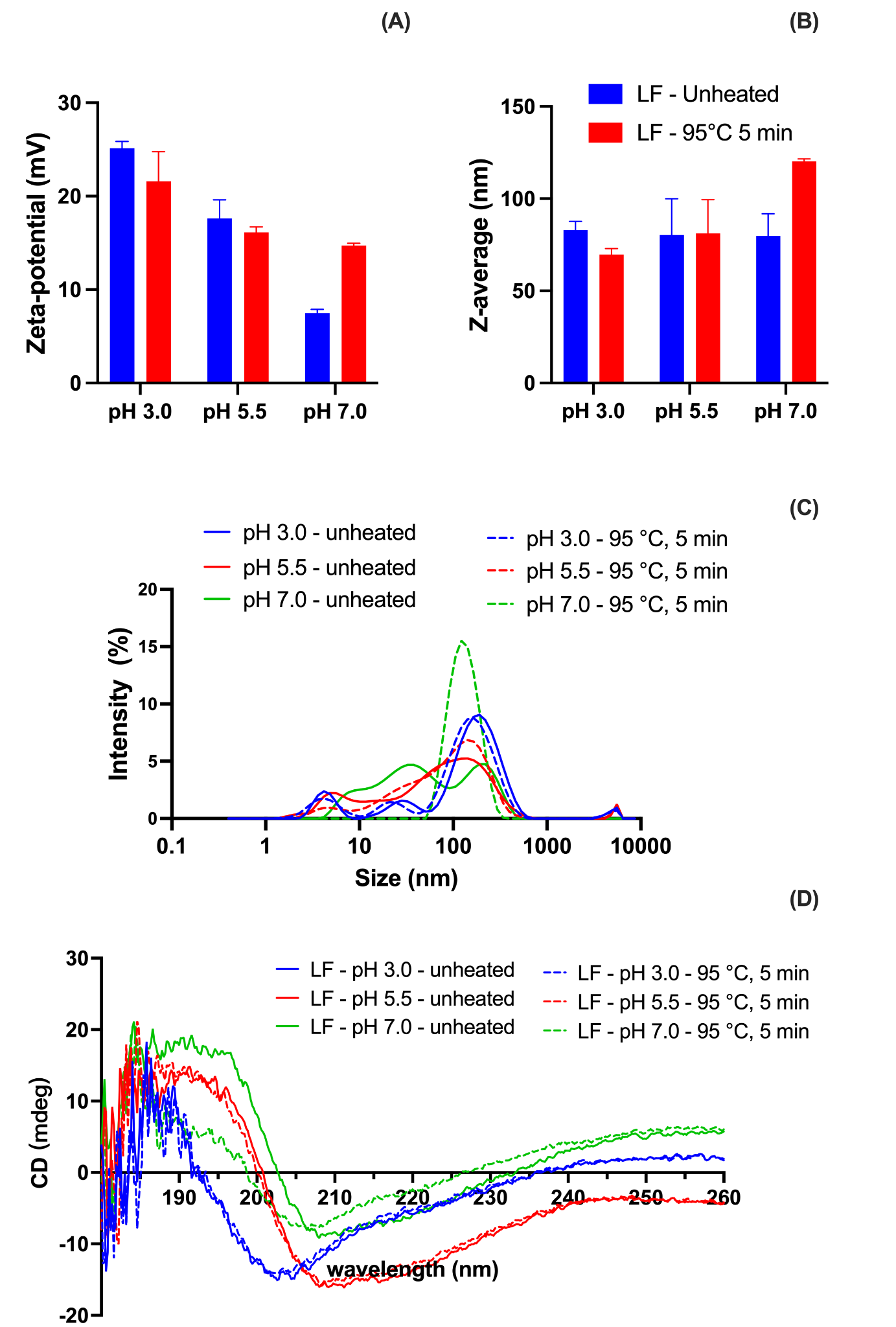


**Figure S11.** Thermal stability of LF. Zeta potential (A), Z-average (B), size distribution (C) and CD spectra (D) of 0.2% LF during thermal treatment under different pH levels.


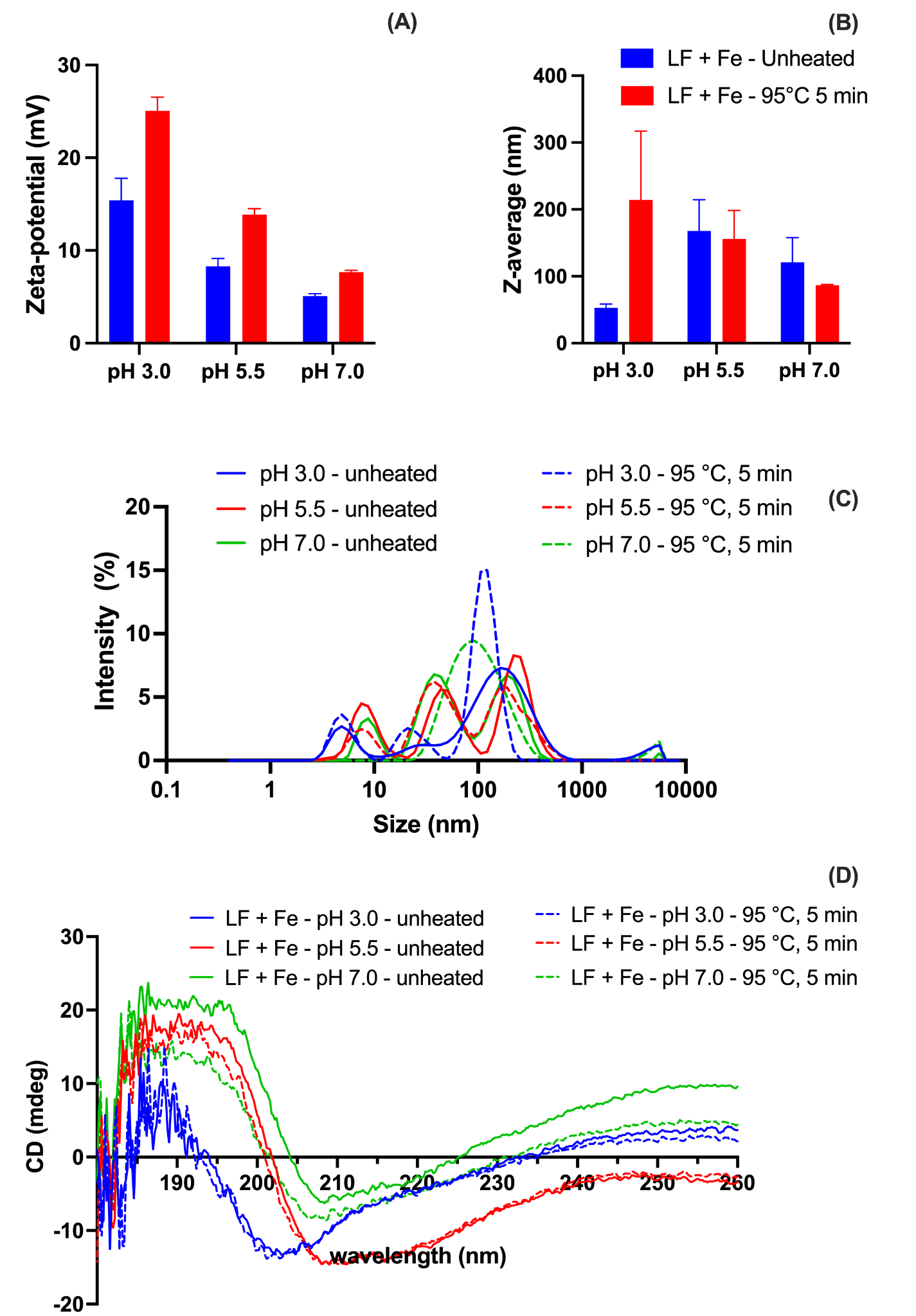


**Figure S12**. Thermal stability of LF+Fe. Zeta potential (A), Z-average (B), size distribution (C) and CD spectra (D) of 0.2% LF+0.4 mM Fe during thermal treatment under different pH levels.


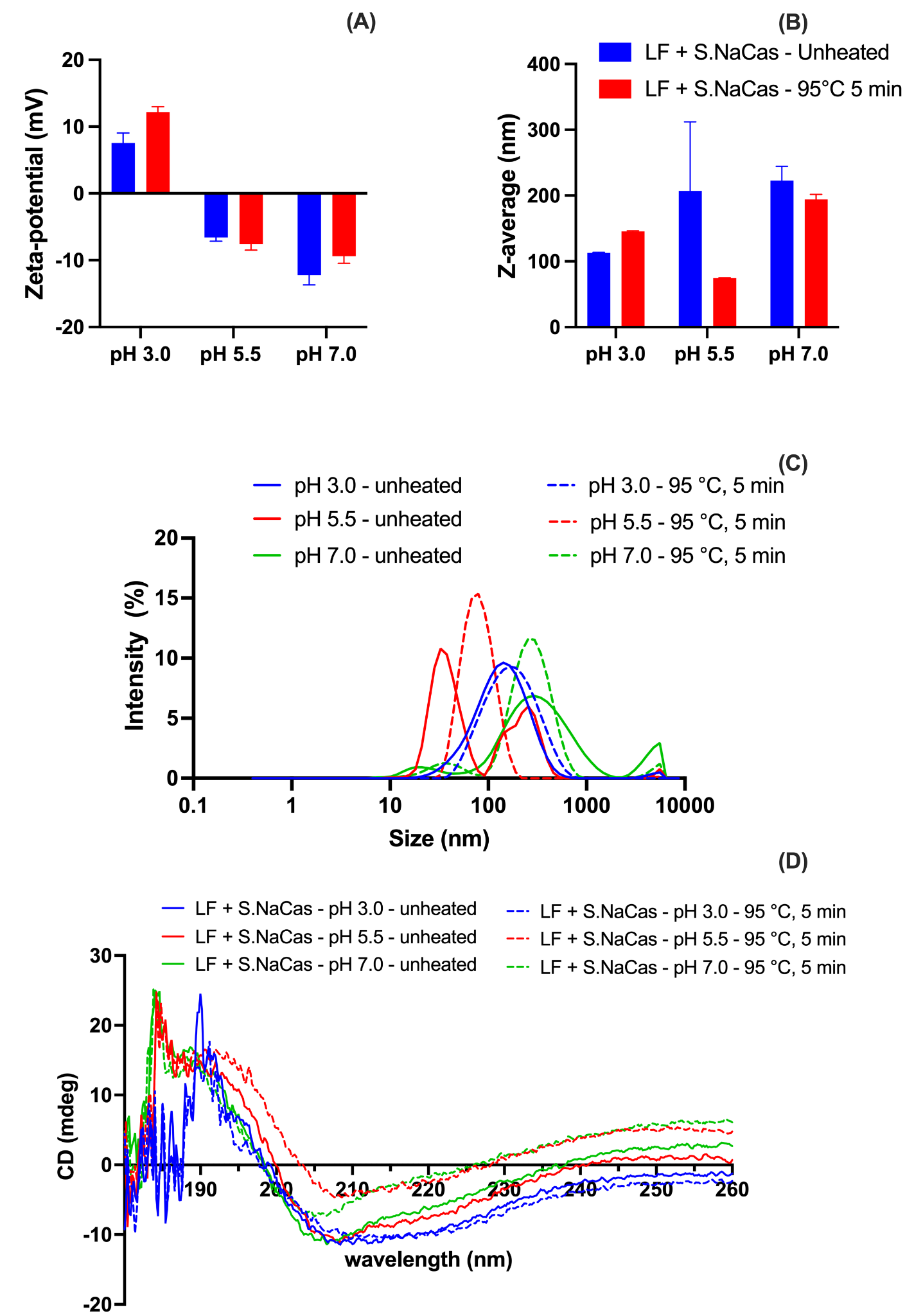


**Figure S13.** Thermal stability of LF+S.NaCas DM. Zeta potential (A), Z-average (B), size distribution (C) and CD spectra (D) of 0.2% LF+S.NaCas 1:1 DM during thermal treatment under different pH levels.


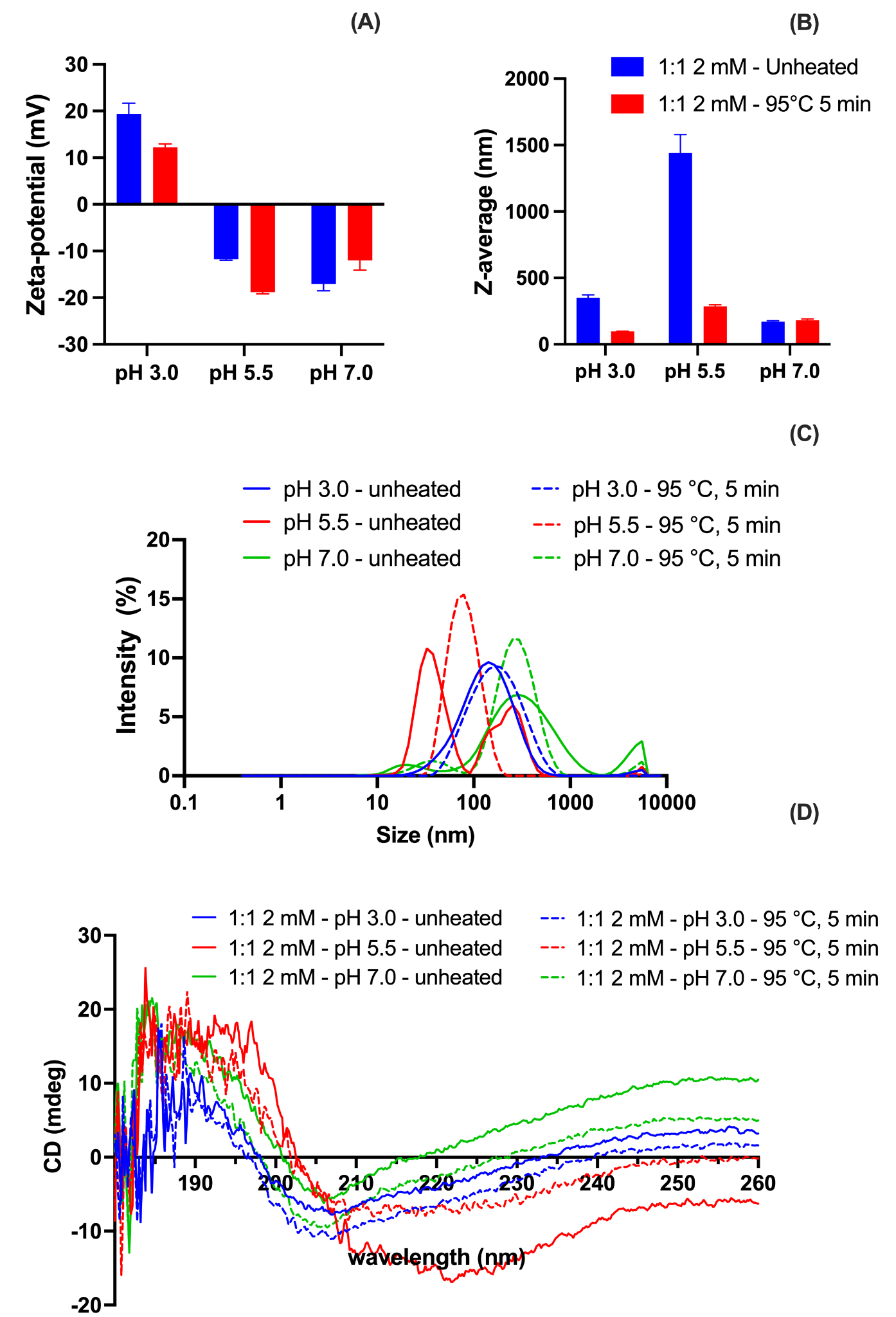


**Figure S14.** Thermal stability of LF-S.NaCas 1:1-Fe 2 mM complex. Zeta potential (A), Z-average (B), size distribution (C) and CD spectra (D) of 0.2% LF-S.NaCas 1:1-Fe 2 mM complex during thermal treatment under different pH levels.

**Table S3.** Simulated CD statistics of secondary structure fraction and structural change of LF and redispersed LF-S.NaCas 1:1-Fe 2 mM complex during thermal treatment at pH 3.0 based on Figure S11-14D.

| pH 3.0 | | Helix | Strand | Turns | Unordered |
| --- | --- | --- | --- | --- | --- |
| LF | Unheated | 0.096 ± 0.017 | 0.315 ± 0.006 | 0.227 ± 0.010 | 0.363 ± 0.021 |
|  | 95°C, 5 min | 0.085 ± 0.008 | 0.333 ± 0.002 | 0.220 ± 0.001 | 0.364 ± 0.005 |
|  | **Change (%)** | **-12.50** | **5.71** | **-3.01** | **0.28** |
| LF+Fe | Unheated | 0.091 ± 0.004 | 0.328 ± 0.002 | 0.227 ± 0.002 | 0.356 ± 0.004 |
|  | 95°C, 5 min | 0.091 ± 0.001 | 0.331 ± 0.002 | 0.224 ± 0.006 | 0.355 ± 0.005 |
|  | **Change (%)** | **1.10** | **0.91** | **-1.32** | **-0.28** |
| LF+S.NaCas  1:1 DM | Unheated | 0.145 ± 0.030 | 0.327 ± 0.030 | 0.211 ± 0.018 | 0.317 ± 0.018 |
|  | 95°C, 5 min | 0.141 ± 0.027 | 0.329 ± 0.022 | 0.218 ± 0.005 | 0.312 ± 0.010 |
|  | **Change (%)** | **-2.76** | **0.30** | **2.84** | **-1.58** |
| LF-S.NaCas-Fe | Unheated | 0.084 ± 0.007 | 0.358 ± 0.008 | 0.210 ± 0.007 | 0.349 ± 0.006 |
|  | 95°C, 5 min | 0.113 ± 0.001 | 0.323 ± 0.003 | 0.223 ± 0.005 | 0.342 ± 0.003 |
|  | **Change (%)** | **34.52** | **-9.78** | **6.19** | **1.72** |

**Table S4.** Simulated CD statistics of secondary structure fraction and structural change of LF and redispersed LF-S.NaCas 1:1-Fe 2 mM complex during thermal treatment at pH 5.5 based on Figure S11-14D.

| pH 5.5 | | Helix | Strand | Turns | Unordered |
| --- | --- | --- | --- | --- | --- |
| LF | Unheated | 0.221 ± 0.008 | 0.274 ± 0.001 | 0.227 ± 0.002 | 0.279 ± 0.010 |
|  | 95°C, 5 min | 0.172 ± 0.021 | 0.316 ± 0.017 | 0.213 ± 0.002 | 0.301 ± 0.005 |
|  | **Change (%)** | **-11.75** | **7.00** | **-8.80** | **8.22** |
| LF+Fe | Unheated | 0.222 ± 0.017 | 0.285 ± 0.020 | 0.22 ± 0.01 | 0.273 ± 0.010 |
|  | 95°C, 5 min | 0.218 ± 0.035 | 0.271 ± 0.043 | 0.23 ± 0.02 | 0.281 ± 0.014 |
|  | **Change (%)** | **-2.10** | **-4.80** | **4.39** | **3.06** |
| LF+S.NaCas  1:1 DM | Unheated | 0.151 ± 0.009 | 0.320 ± 0.012 | 0.212 ± 0.002 | 0.318 ± 0.001 |
|  | 95°C, 5 min | 0.086 ± 0.008 | 0.383 ± 0.013 | 0.190 ± 0.006 | 0.341 ± 0.000 |
|  | **Change (%)** | **-43.19** | **19.87** | **-10.16** | **7.23** |
| LF-S.NaCas-Fe | Unheated | 0.070 ± 0.004 | 0.383 ± 0.004 | 0.225 ± 0.006 | 0.323 ± 0.005 |
|  | 95°C, 5 min | 0.061 ± 0.000 | 0.387 ± 0.001 | 0.224 ± 0.000 | 0.329 ± 0.001 |
|  | **Change (%)** | **-12.86** | **0.91** | **-0.44** | **1.86** |

**Table S5.** Simulated CD statistics of secondary structure fraction and structural change of LF and redispersed LF-S.NaCas 1:1-Fe 2 mM complex during thermal treatment at pH 7.0 based on Figure S11-14D.

| pH 7.0 | | Helix | Strand | Turns | Unordered |
| --- | --- | --- | --- | --- | --- |
| LF | Unheated | 0.129 ± 0.015 | 0.360 ± 0.020 | 0.181 ± 0.006 | 0.330 ± 0.001 |
|  | 95°C, 5 min | 0.070 ± 0.019 | 0.372 ± 0.029 | 0.196 ± 0.010 | 0.362 ± 0.007 |
|  | **Change (%)** | **-47.22** | **4.69** | **7.50** | **9.91** |
| LF+Fe | Unheated | 0.093 ± 0.013 | 0.398 ± 0.010 | 0.164 ± 0.005 | 0.346 ± 0.008 |
|  | 95°C, 5 min | 0.112 ± 0.014 | 0.357 ± 0.007 | 0.193 ± 0.006 | 0.338 ± 0.014 |
|  | **Change (%)** | **20.43** | **-10.30** | **17.74** | **-2.17** |
| LF+S.NaCas 1:1 DM | Unheated | 0.089 ± 0.006 | 0.348 ± 0.004 | 0.232 ± 0.004 | 0.332 ± 0.000 |
|  | 95°C, 5 min | 0.095 ± 0.004 | 0.344 ± 0.006 | 0.235 ± 0.000 | 0.327 ± 0.010 |
|  | **Change (%)** | **6.78** | **-1.15** | **1.51** | **-1.51** |
| LF-S.NaCas-Fe | Unheated | 0.092 ± 0.011 | 0.351 ± 0.008 | 0.224 ± 0.003 | 0.333 ± 0.008 |
|  | 95°C, 5 min | 0.092 ± 0.000 | 0.340 ± 0.008 | 0.234 ± 0.005 | 0.335 ± 0.004 |
|  | **Change (%)** | **2.17** | **-5.27** | **3.56** | **3.04** |


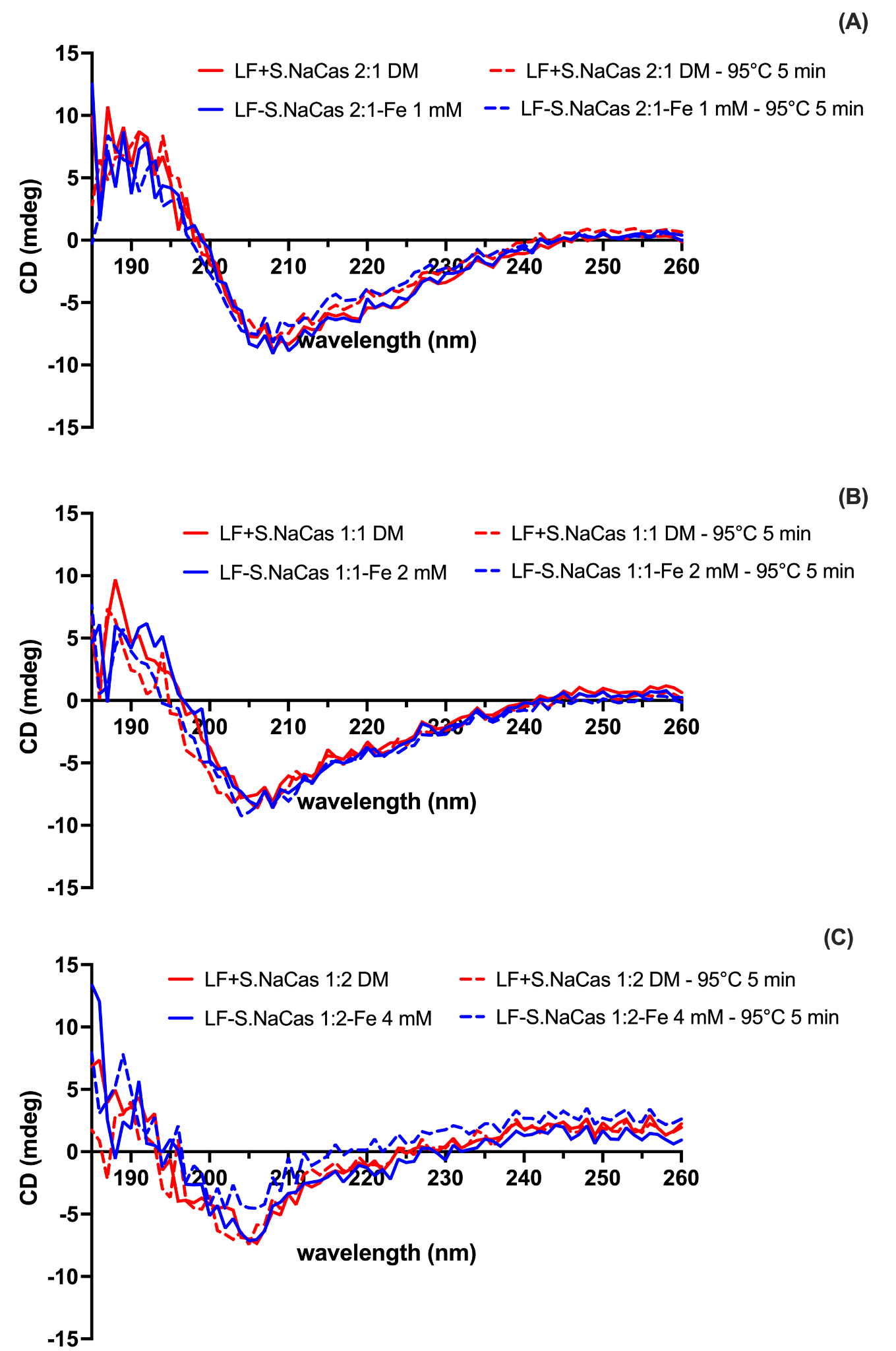


**Figure S15**. CD spectra of different ratios of LF + S.NaCas direct mixture and redispersed LF-S.NaCas-Fe mM complex before and after thermal treatment (A-C) under pH 7.0. All protein were diluted to 0.02 mg/mL before CD measurement.

**Table S6.** Simulated CD statistics of secondary structure fraction and structural change of LF+S.NaCas DM and redispersed LF-S.NaCas -Fe mM complex during thermal treatment at pH 7.0 based on Fig 12.

|  | | Helix | Strand | Turns | Unordered |
| --- | --- | --- | --- | --- | --- |
| 2:1 DM | Unheated | 0.104 ± 0.012 | 0.347 ± 0.001 | 0.221 ± 0.003 | 0.328 ± 0.013 |
|  | 95°C, 5 min | 0.104 ± 0.016 | 0.346 ± 0.019 | 0.227 ± 0.008 | 0.324 ± 0.004 |
|  | **Change (%)** | **0.48** | **-0.43** | **2.49** | **-1.22** |
| 2:1 1 mM | Unheated | 0.108 ± 0.006 | 0.341 ± 0.016 | 0.222 ± 0.013 | 0.330 ± 0.004 |
|  | 95°C, 5 min | 0.090 ± 0.018 | 0.351 ± 0.007 | 0.230 ± 0.002 | 0.329 ± 0.014 |
|  | **Change (%)** | **-16.67** | **2.93** | **3.61** | **-0.30** |
| 1:1 DM | Unheated | 0.089 ± 0.006 | 0.348 ± 0.004 | 0.232 ± 0.004 | 0.332 ± 0.000 |
|  | 95°C, 5 min | 0.095 ± 0.004 | 0.344 ± 0.006 | 0.235 ± 0.000 | 0.327 ± 0.010 |
|  | **Change (%)** | **6.78** | **-1.15** | **1.51** | **-1.51** |
| 1:1 2 mM | Unheated | 0.092 ± 0.011 | 0.351 ± 0.008 | 0.224 ± 0.003 | 0.333 ± 0.008 |
|  | 95°C, 5 min | 0.092 ± 0.000 | 0.340 ± 0.008 | 0.234 ± 0.005 | 0.335 ± 0.004 |
|  | **Change (%)** | **2.17** | **-5.27** | **3.56** | **3.04** |
| 1:2 DM | Unheated | 0.051 ± 0.002 | 0.396 ± 0.006 | 0.209 ± 0.006 | 0.346 ± 0.002 |
|  | 95°C, 5 min | 0.054 ± 0.004 | 0.377 ± 0.005 | 0.217 ± 0.001 | 0.352 ± 0.010 |
|  | **Change (%)** | **6.93** | **-4.80** | **4.08** | **1.88** |
| 1:2 4 mM | Unheated | 0.054 ± 0.014 | 0.383 ± 0.020 | 0.219 ± 0.014 | 0.343 ± 0.010 |
|  | 95°C, 5 min | 0.053 ± 0.004 | 0.376 ± 0.001 | 0.222 ± 0.001 | 0.350 ± 0.006 |
|  | **Change (%)** | **0.00** | **-1.83** | **1.06** | **1.36** |

**
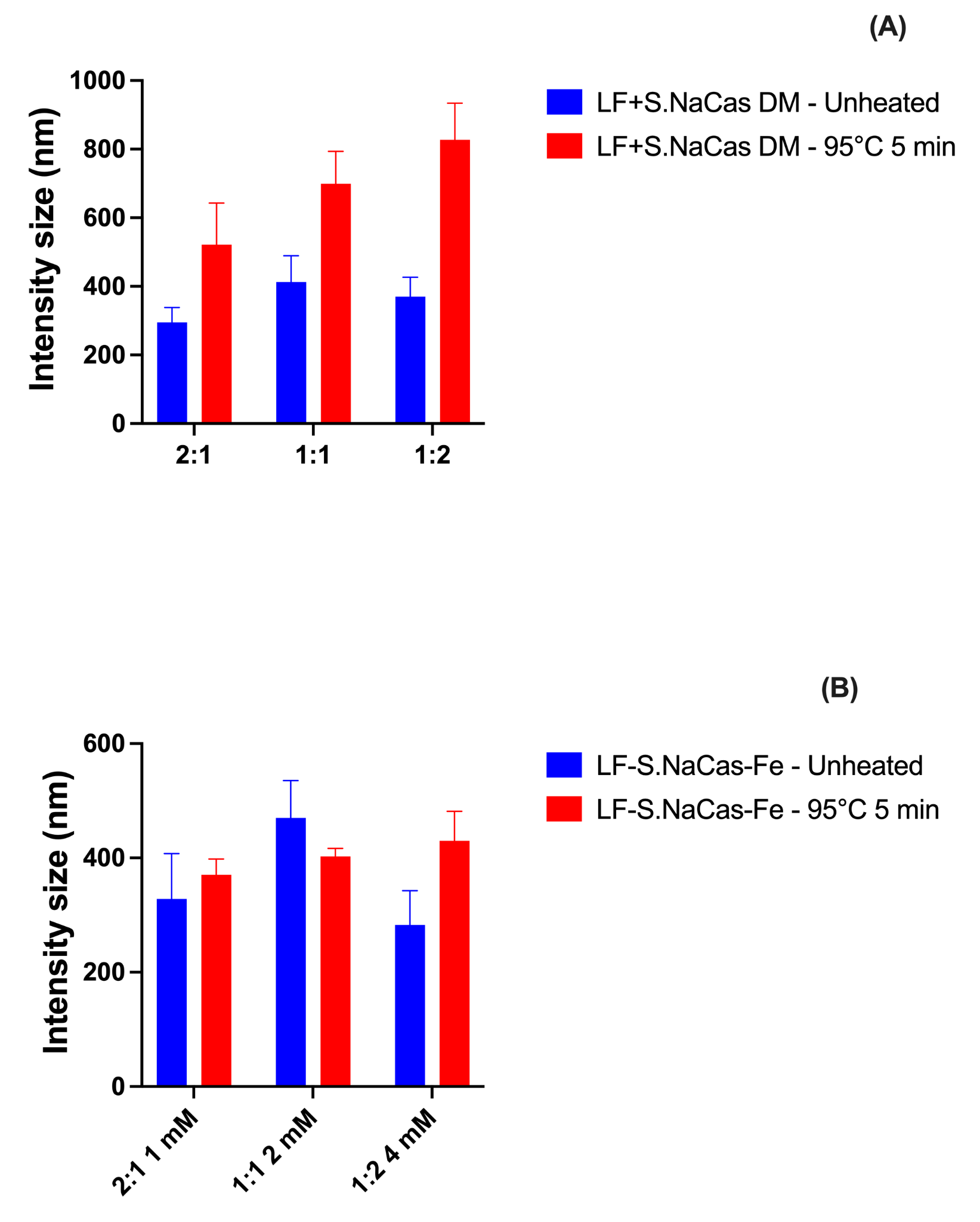
**

**Figure S16.** Intensity particle size of LF + S.NaCas direct mixture (A) and redispersed LF-S.NaCas-Fe complex (B) before and after thermal treatment at pH 7.0.
